# Age‐related qualitative differences in post‐error cognitive control adjustments

**DOI:** 10.1111/bjdp.12403

**Published:** 2022-01-18

**Authors:** Mirela Dubravac, Claudia M. Roebers, Beat Meier

**Affiliations:** ^1^ Institute of Psychology University of Bern Bern Switzerland

**Keywords:** cognitive conflict, cognitive control, cognitive development, flanker task, post‐error slowing, Simon task, Stroop task

## Abstract

Detecting an error signals the need for increased cognitive control and behavioural adjustments. Considerable development in performance monitoring and cognitive control is evidenced by lower error rates and faster response times in multi‐trial executive function tasks with age. Besides these quantitative changes, we were interested in whether qualitative changes in balancing accuracy and speed contribute to developmental progression during elementary school years. We conducted two studies investigating the temporal and developmental trajectories of post‐error slowing in three prominent cognitive conflict tasks (Stroop, Simon, and flanker). We instructed children (8‐, 10‐, and 12‐year‐old) and adults to respond as fast and as accurately as possible and measured their response times on four trials after correct and incorrect responses to a cognitive conflict. Results revealed that all age groups had longer response times on post‐error versus post‐correct trials, reflecting post‐error slowing. Critically, slowing on the first post‐error trial declined with age, suggesting an age‐related reduction in the orienting response towards errors. This age effect diminished on subsequent trials, suggesting more fine‐tuned cognitive control adjustments with age. Overall, the consistent pattern across tasks suggests an age‐related change from a relatively strong orienting response to more balanced cognitive control adaptations.

**Statement of contribution**

***What is already known?***

- After errors, children slow down more strongly than adults.
- Post‐error slowing decreases with time.

***What does this study add?***

- The temporal trajectory of post‐error slowing changes from childhood to adulthood with the strongest age effects on the first post‐error trial.
- The temporal and developmental trajectories generalize across three cognitive conflict tasks.

## INTRODUCTION

Executive functions refer to a diverse set of processes involved in the regulation of goal‐oriented behaviour. Already, young children show executive functioning as they switch between different tasks, inhibit inappropriate actions, and adjust behaviour flexibly to changing demands. However, much development is still taking place during childhood, as evidenced by striking differences between children’s and adults’ performance on executive function tasks (Davidson et al., 2006; Ridderinkhof & van der Molen, 1995). Usually, children need more time to respond and commit more errors than adults. The developmental trajectory of these quantitative performance improvements depends on the demands that a specific task poses on different aspects of executive functioning, complicating the understanding of emerging ‘executive control’ (Anderson, 2002; Davidson et al., 2006; Diamond, 2013). Thus, the question arises of how children achieve to orchestrate different cognitive processes to work in concert for optimal performance. Recent theoretical advances point to qualitative changes in the coordination of performance monitoring and cognitive control as a driving force for developmental progression (Chevalier, 2015; Hämmerer et al., 2014; Roebers, 2017). Adopting a fine‐grained approach to the study of developing executive functioning, this study investigated the developmental trajectories of processes involved in monitoring and adjusting response times on a trial‐by‐trial basis.

Trial‐by‐trial adjustments are already evident in young children as they show longer response times after cognitive conflicts (Ambrosi et al., 2016; Grundy & Keyvani Chahi, 2017; Smulders et al., 2018). Similarly, detecting self‐generated errors leads also to longer response times on subsequent trials (Brewer & Smith, 1989; Danielmeier & Ullsperger, 2011; Dubravac et al., 2020; Fairweather, 1978; Gupta et al., 2009; Jones et al., 2003; King et al., 2010; McDermott et al., 2007; Schroder et al., 2020; Smulders et al., 2016; Thaqi & Roebers, 2020). Post‐error slowing has been attributed to motor inhibition (Marco‐Pallarés et al., 2008; Ridderinkhof, 2002), inhibition of irrelevant information (King et al., 2010), delayed processing of sensory information (Buzzell et al., 2017; Laming, 1979), attentional orienting towards the source of the error (Notebaert et al., 2009), and cognitive control adjustments following error detection (Botvinick et al., 2001). These accounts are complementary, as motor inhibition facilitates attentional orientation towards the error delaying information processing and thus gaining time for the implementation of cognitive control processes, such as response threshold adjustments (Danielmeier & Ullsperger, 2011; Wessel, 2018). That is, error detection triggers different processes that operate at different time scales and may follow distinct developmental trajectories (Compton et al., 2017; Dubravac et al., 2020; Dudschig & Jentzsch, 2009; Jentzsch & Dudschig, 2009; Van der Borght et al., 2016).

Brewer and Smith (1989) were among the first to investigate developmental changes in post‐error response times across multiple trials. The participants of a wide age range (5–15 years and adults) performed a simple choice response time task. The examination of correct and error response times for error‐to‐error sequences varying from 2 to 16 trials revealed more fine‐tuned adjustments with age as variability in response times decreased (Brewer & Smith, 1989). More recent studies confirmed the age‐related decrease in response time variability surrounding errors and, particularly, post‐error slowing (Dubravac et al., 2020; Gupta et al., 2009; Smulders et al., 2016; Thaqi & Roebers, 2020). The decrease in post‐error slowing between 7‐ and 8‐year‐old children and between 9‐ and 10‐year‐old children was related to the development of inhibitory control in this age range (Gupta et al., 2009). This suggests that developing inhibitory control may promote smoother response time adjustments after errors.

The age range between 8 years and young adulthood represents a transition period associated with qualitative changes in cognitive control (Chevalier & Blaye, 2016; Chevalier et al., 2013, 2015; Munakata et al., 2012). Children transition from relying predominantly on environmental cues signalling increased cognitive control demands to relying on self‐generated error detection signals originating from internal performance monitoring. Furthermore, children become more and more efficient in coordinating different cognitive control modes according to moment‐to‐moment variations in task demands and metacognitive performance monitoring outcomes (Chevalier, 2015; Chevalier & Blaye, 2016; Chevalier et al., 2015; Czernochowski, 2014; Roebers, 2017). Considering differences in the developmental trajectories of distinct components of executive functions, as inhibitory control or cognitive flexibility (Anderson, 2002; Davidson et al., 2006; Diamond, 2013), the age range between 8 years and adulthood is an interesting period for investigating fine‐grained qualitative changes in post‐error response time adjustments.

Building on previous work, we set up two studies investigating developmental and temporal trajectories of post‐error slowing in three different executive function tasks (Simon, Stroop, and flanker). The first study compared the Stroop and Simon tasks. The second study compared the Stroop and flanker tasks. The participants of four age groups (8‐, 10‐, 12‐year‐olds, and young adults) took part in either one of the two studies. The samples differed between studies, and the Stroop task was administered in both samples to have an internal replication that helps to disentangle true differences between tasks from sampling effects. All three tasks elicit cognitive conflict on incongruent trials, where irrelevant stimulus dimensions (in the Stroop and Simon tasks) or irrelevant distractors (in the flanker task) trigger a prepotent but incorrect response that has to be inhibited to respond correctly. To induce errors experimentally and to have a considerable number of errors, we presented incongruent trials less frequently than congruent trials (Gratton et al., 1992; Stürmer et al., 2002). Thus, every fifth trial was an incongruent trial. Response times on four subsequent congruent trials were measured as a function of the correctness of the incongruent trial. By subtracting response times following correct conflict trials from response times following incorrect conflict trials, conflict‐related slowing was subtracted from conflict + error‐related slowing. The difference thus represents pure post‐error slowing (Dubravac et al., 2020). We expected substantial slowing after errors in all age groups and tasks.

Previous studies indicated an age‐related decrease in response time variability, and in line with the theory of developing cognitive control (Brewer & Smith, 1989; Davidson et al., 2006; Diamond, 2013), we predicted an age‐related decrease in post‐error slowing. With respect to the time course of post‐error slowing, we hypothesized that slowing would decrease across trials. The strongest slowing is expected on the first post‐error trial reflecting short‐lived attentional distraction (Notebaert et al., 2009). Less pronounced but persistent slowing across subsequent trials, in contrast, would indicate strategic cognitive control adjustments in the sense of increased caution implemented by elevated response thresholds (Botvinick et al., 2001; Dutilh et al., 2012). By measuring slowing on four post‐error trials, we aimed to track the dynamics of these component processes underlying post‐error slowing.

The main aim of this study was to identify age differences in the component processes underlying post‐error slowing. Based on theories assuming increasing attentional control and response inhibition, and more optimal coordination of cognitive control with age (Anderson, 2002; Chevalier, 2015; Diamond, 2013; Luna et al., 2010; Luna & Sweeney, 2004), we hypothesized that the time course of post‐error slowing would vary as a function of age. This would be evident in an interaction between age group and post‐error trial. Specifically, we hypothesized that the younger age groups would have more difficulties inhibiting attentional distraction from an error resulting in a strong attentional orienting response towards the error on the first post‐error trial. With increasing attentional control, slowing on the first post‐error trial should decrease with age. As slowing on subsequent trials reflects more strategic cognitive control adjustments, we expected this kind of slowing in adults and to a lesser degree in children.

## METHOD

### Participants

The children were recruited from local schools, and the young adults were undergraduate psychology students. The ethics committee of the local University approved the studies. Prior to testing the participants, we obtained written informed consent of the adult participants and children's parents. Children received a small present for their participation, and adults received credits.

### Task structure and analysis

**FIGURE 1:**
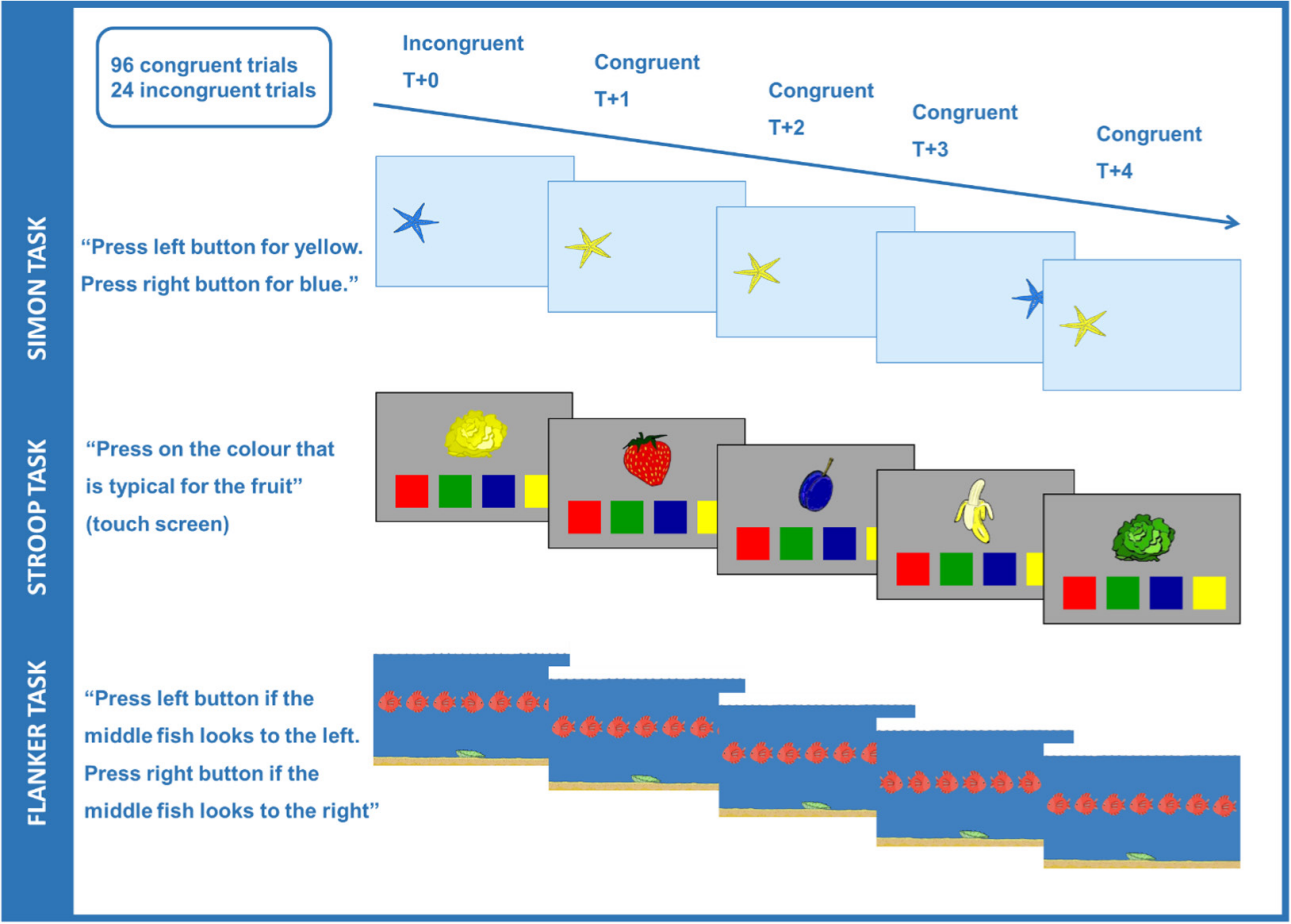
Schematic depiction of the three tasks

In the Stroop task, the colour of fruit and vegetables either matched their colour in the real‐world (congruent) or was an unrealistic colour (incongruent), and the task was to indicate the real‐world colour. In the Simon task, the participants had to indicate the colour of a starfish by pressing a button either with their right or left hand. The presentation side of the starfish was either congruent with the required response side (i.e., left side presentation + left hand response) or was incongruent (i.e., left side presentation + right hand response). In the flanker task, a central target fish was presented among distracting fish that faced either in the same (congruent) or different (incongruent) direction, and the task was to respond according to the side the central fish was facing. All tasks comprised a pure congruent block and a critical mixed block. The mixed block comprised 24 incongruent trials evenly interspersed among 96 congruent trials (Meier et al., 2009; Woodward et al., 2003). Every fifth trial was thus incongruent. Figure 1 gives an overview of the three tasks.

As a manipulation check, we analysed the congruency effect and the distribution of error rates across the participants (cf. Supplementary Material). The main analysis applied to response times on four congruent trials as a function of the correctness of the response on the previous incongruent trial. First, we tested the hypothesis of slower responses after errors for each age group separately by conducting one‐sided paired *t*‐tests comparing post‐error response times to post‐correct response times on each of the four trials. This gave us a first impression of the persistence of the post‐error slowing effect for each age group. Next, we created the main dependent variable *post*‐*error slowing* by subtracting post‐correct response times from post‐error response times. This difference score was subjected to an analysis of variance (ANOVA) with the within‐subject factor trial (T + 1, T + 2, T + 3, T + 4) and the between‐subject factor age group (8‐year‐olds, 10‐year‐olds, 12‐year‐olds, and adults). An alpha level of .05 was set. Where appropriate, we report Greenhouse–Geisser corrected values for *F*‐tests and applied the Bonferroni correction for multiple comparisons in *post‐hoc* tests.

## STUDY 1

### Participants

We recruited 44 8‐year‐olds, 46 10‐year‐olds, 52 12‐year‐olds, and 40 young adults. Due to technical problems, we did not obtain Stroop task data from one 8‐year‐old, five 10‐year‐olds, and three 12‐year‐olds. After data screening, we applied the following task‐wise exclusion criteria. We excluded the participants who did not commit any error on the critical incongruent trials intended to elicit errors (Stroop task: nine 8‐year‐olds, seven 10‐year‐olds, eleven 12‐year‐olds, and 17 adults; Simon task: three 8‐year‐olds, six 10‐year‐olds, six 12‐year‐olds, and five adults). We also excluded the participants with an accuracy rate under 50% (Stroop task: one 8‐year‐old; Simon task: two 10‐year olds, one 12‐year‐old, and one adult). This is important because low accuracy rates indicate that they probably did not understand the task, and then they would not be aware of their errors. As error rates influence post‐error slowing (Steinborn et al., 2012), we further excluded the participants with error rates higher than two times the standard deviation (Stroop task: one 8‐year‐old, one 10‐year‐old, two 12‐year‐olds; Simon task: two 8‐year‐olds, one 10‐year‐old, and two 12‐year‐olds). This resulted in a more homogenous sample and allowed comparisons across the participants, tasks, and groups. Finally, because we included only correct trials in the analysis of post‐error response times, the participants who committed errors on post‐error trials (i.e., double errors) were also excluded as they did not provide values in those critical cells (Stroop task: no one; Simon task: one 12‐year‐old and one adult). Table 1 presents the demographic characteristics of the final sample.

**TABLE 1:** Study 1: Demographic characteristics of the sample

Age group	Stroop task					Simon task				
	*M*_Age_	*SD*_Age_	Age range	Female/Male	*n*	*M*_Age_	*SD*_Age_	Age range	Female/Male	*n*
8‐year‐olds	8.4	0.4	7.8–8.9	10/22	32	8.4	0.4	7.8–9.0	14/25	39
10‐year‐olds	10.1	0.4	9.4–10.9	13/20	33	10.1	0.4	9.4–10.9	16/21	37
12‐year‐olds	12	0.4	11.2–13	16/20	36	12	0.4	11.2–13.0	19/23	42
Adults	23	3.6	19.5–34.9	17/6	23	22.9	3.2	19.5–34.9	28/5	33

### Material

The Stroop task was adapted from Archibald and Kerns (1999). The stimuli were coloured quadrants and drawings of salad, strawberry, plum, and banana appearing in the middle of a tablet screen. Below were quadrants displayed in red, green, blue, and yellow. The Simon task was the same as in a previous study (Dubravac et al., 2020). The stimuli were yellow and blue starfish appearing on the left or right side of a laptop screen. Stimuli are presented in Figure 1.

### Procedure

The procedure was the same for children and adults. The participants were tested individually and completed first the Stroop task on a tablet and then the Simon task on a laptop computer. The tablet and laptop were placed at arm length distance, and the response was given by pressing on the touch screen of the tablet (Stroop task) or by pressing on either the left or right mouse button of the laptop (Simon task). The participants were asked to respond as accurately and as fast as possible.

#### Stroop task

The Stroop task consisted of three blocks with a fixed block order, which was not repeated. The first block comprised 12 trials, the second block 24 trials, and the third block 120 trials. In the first block, coloured quadrants were presented in the middle of the screen, and the participants had to choose the corresponding colour from four alternatives presented below the probe colour quadrant. They gave the response by pressing directly on the touchscreen on the colour quadrant. In the second, purely congruent block, fruit were presented in the congruent colour (i.e., typical colour of the fruit). The participants had to choose the corresponding colour from four alternatives presented below the probe colour fruit. The third block was the critical mixed block, in which fruit were either presented in the congruent colour or in one of the three incongruent colours (i.e., not a typical colour of the fruit). The participants had to press on the colour that is typical for the probe fruit (i.e., yellow for banana). On 96 trials, the colour was congruent, and on 24 trials, the colour was incongruent. The incongruent trials were determined randomly with replacement and were evenly interspersed among the 96 congruent trials, occurring on every fifth trial. Each trial started with a fixation cross for 250 ms (= response‐stimulus interval) in the middle of the screen, followed by the probe stimulus (i.e., coloured quadrant in the first block or fruit in the second and third blocks) which stayed on screen until response. To ensure that the task was clear to the participants, four practice trials were included before the critical third block. In the case of more than two errors, the examiner explained the task again, and another four practice trials were presented. After a successful practice run, the mixed block started. The mixed block was preceded by four congruent warm‐up trials not included in the analysis.

#### Simon task

The Simon task consisted of two blocks with a fixed block order, which was not repeated. The first block comprised 24 trials, and the second block 120 trials. The participants responded by pressing on the left or right mouse button of the laptop keyboard. For yellow starfish, the participants had to press the left mouse button with the left index finger. For blue starfish, the participants had to press the right mouse button with the right index finger. In the first, purely congruent block, 12 yellow and 12 blue starfish appeared in random order always on the congruent response side. The second block was the critical mixed block, in which the starfish were either presented on the congruent or incongruent side. The 24 incongruent trials were determined randomly with replacement and were evenly interspersed among the 96 congruent trials, occurring on every fifth trial. Each trial consisted of a fixation cross for 250 ms (= response‐stimulus interval) in the middle of the screen, followed by a yellow or blue starfish, which appeared either on the left or right side and stayed on screen until response. To ensure that the task was clear to the participants, the examiner showed two congruent example trials before the congruent practice block and two incongruent example trials before the mixed practice block. The practice blocks consisted of four practice trials. In the case of more than two errors in the practice block, the examiner explained the task again, and another four practice trials were presented. After a successful practice run, the respective block started. The mixed block was preceded by four congruent warm‐up trials not included in the analysis.

### Results

#### Stroop task

**FIGURE 2:**
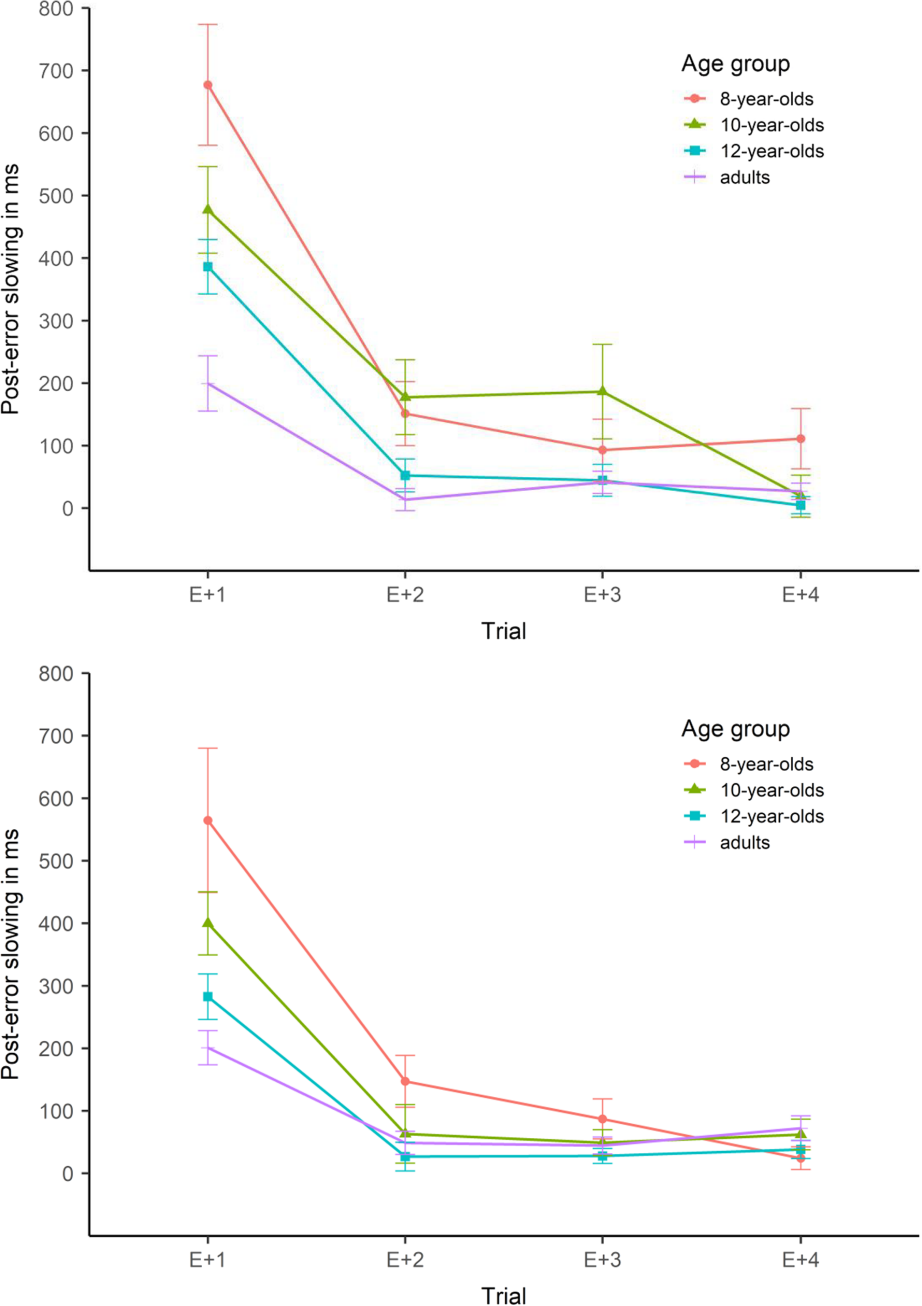
Study 1: Trajectories of post‐error slowing in the Stroop task (upper panel) and Simon task (lower panel). *Note*. Error bars show standard errors

The average number of errors was 2.7 (*SE* = 0.2) for 8‐year‐olds, 2.3 (*SE* = 0.3) for 10‐year‐olds, 2.6 (*SE* = 0.2) for 12‐year‐olds, and 1.7 (*SE* = 0.3) for adults. Table 2 shows response times on correct and incorrect incongruent trials (T + 0) and the subsequent trials (T + 1, T + 2, T + 3, and T + 4). Figure 2 depicts the trajectories of post‐error slowing. The 4 × 4 ANOVA on post‐error slowing revealed a main effect of age group, *F*(3, 120) = 8.80, *p* < .001, $\eta_{p}^{2}$ = .18, and a main effect of trial, *F*(2.53, 303.40) = 52.58, *p* < .001, $\eta_{p}^{2}$ = .30. The interaction was also significant, *F*(7.59, 303.40) = 2.89, *p* = .005, $\eta_{p}^{2}$ = .07. To resolve the interaction, we compared the age groups on each trial. Separate one‐way ANOVAs revealed a significant main effect of age group on the first post‐error trial, *F*(3, 120) = 7.37, *p* < .001, $\eta_{p}^{2}$ = .16, as well as on the second post‐error trial, *F*(3, 120) = 2.87, *p* = .039, $\eta_{p}^{2}$ = .07, but not on the third and fourth trials, *F*(3, 120) = 1.84, *p* = .144, $\eta_{p}^{2}$ = .04, and *F*(3, 120) = 2.35, *p* = .075, $\eta_{p}^{2}$ = .06, respectively. Comparing the age groups on T + 1 revealed an age‐related decrease in post‐error slowing (8 = 10, 8 > 12, 8 > adults; 10 = 12, 10 = adults; 12 = adults). *Post‐hoc* comparisons were not significant for T + 2, though.

**TABLE 2:** Study 1: Response times in ms on incongruent conflict trials (T + 0) and subsequent correct congruent trials (T + 1, T + 2, T + 3, and T + 4) as a function of age group and trial type (correctness on T + 0)

Age group	Trial type	T + 0		T + 1		T + 2		T + 3		T + 4	
		*M*	*SE*	*M*	*SE*	*M*	*SE*	*M*	*SE*	*M*	*SE*
*Stroop task*											
8‐year‐olds	Correct	1514	43	1082	38	1010	38	974	33	943	35
	Error	1127	56	1759	109	1161	68	1067	65	1054	56
	Difference	−387	**	677	**	151	*	93	*	111	*
10‐year‐olds	Correct	1276	33	886	24	827	20	804	15	789	18
	Error	1032	51	1363	68	1004	64	991	80	808	37
	Difference	−244	**	477	**	177	*	187	*	19	
12‐year‐olds	Correct	1093	37	749	21	720	18	704	17	708	19
	Error	833	33	1135	49	772	34	749	34	713	25
	Difference	−260	**	386	**	52	*	45	*	5	
Adults	Correct	804	16	586	10	581	9	576	9	570	10
	Error	649	32	786	45	594	20	617	17	597	14
	Difference	−155	**	200	**	13		41	*	27	*
*Simon task*											
8‐year‐olds	Correct	965	29	835	26	748	24	725	20	712	22
	Error	629	29	1399	122	896	46	812	38	736	27
	Difference	−336	**	564	**	148	**	87	*	24	
10‐year‐olds	Correct	786	15	684	16	614	18	592	16	578	14
	Error	513	34	1084	59	677	52	642	31	640	32
	Difference	−273	**	400	**	63		50	*	62	*
12‐year‐olds	Correct	638	14	557	12	532	13	496	11	505	12
	Error	435	15	840	40	559	24	524	16	544	19
	Difference	−203	**	283	**	27		28	*	39	*
Adults	Correct	501	10	425	8	391	9	388	8	376	9
	Error	340	11	626	30	440	25	432	17	448	26
	Difference	−161	**	201	**	49	*	44	*	72	**

Note

Significant differences (error – correct) are marked with **p < .001, *p < .05.

#### Simon task

The average number of errors was 6.2 (*SE* = 0.6) for 8‐year‐olds, 6.3 (*SE* = 0.7) for 10‐year‐olds, 5.4 (*SE* = 0.5) for 12‐year‐olds, and 3.8 (*SE* = 0.5) for adults. Table 2 shows response times on correct and incorrect incongruent trials (T + 0) and the subsequent trials (T + 1, T + 2, T + 3, and T + 4). Figure 2 depicts the trajectories of post‐error slowing. The 4 × 4 ANOVA on post‐error slowing revealed a main effect of age group, *F*(3, 147) = 4.80, *p* = .003, $\eta_{p}^{2}$ = .09, and a main effect of trial, *F*(1.58, 231.86) = 64.03, *p* < .001, $\eta_{p}^{2}$ = .30. The interaction was also significant, *F*(4.73, 231.86) = 3.88, *p* = .003, $\eta_{p}^{2}$ = .07. To resolve the interaction, we compared the age groups on each trial. Separate one‐way ANOVAs revealed a significant main effect of age group on the first post‐error trial, *F*(3, 147) = 5.18, *p* = .002, $\eta_{p}^{2}$ = .10, but not on the following trials, *F*(3, 147) = 2.37, *p* = .073, $\eta_{p}^{2}$ = .05, *F*(3, 147) = 1.44, *p* = .233, $\eta_{p}^{2}$ = .03, and *F*(3, 147) = 1.24, *p* = .297, $\eta_{p}^{2}$ = .02, respectively. Comparing the age groups on T + 1 revealed an age‐related decrease in post‐error slowing (8 = 10, 8 > 12, 8 > adults; 10 = 12, 10 = adults; 12 = adults).

### Discussion

The participants of four age groups (8‐, 10‐, 12‐year‐old children, and young adults) performed the Stroop and Simon tasks with the instruction to respond as fast and accurately as possible. Post‐error slowing was measured as the difference in response times on four trials following an error versus a correct response. As predicted, reliable slowing was found in both tasks and post‐error slowing decreased with age. Age‐related differences were most pronounced on the first post‐error trial, with a relatively continuous decrease from 8 years to young adulthood. In line with our hypothesis of decreasing post‐error slowing across trials, the extent of slowing decreased sharply from the first to the second trial, more so for the younger age groups. This was reflected in an interaction between age group and trial, confirming our hypothesis of age‐related differences in the time courses of post‐error slowing.

Compared to adults, children showed a steeper decrease in slowing from the first to the second trial, abolishing the age effect on the second trial in the Simon task. Although the age effect was still significant on the second trial in the Stroop task, *post‐hoc* comparisons yielded no significant differences between age groups suggesting that this age effect is negligible after the first trial. Before discussing task differences, we first present Study 2, where different participants performed the same Stroop task and a flanker task. Replicating the Stroop task results and extending the results to the flanker task will provide a better basis for discussing task‐related differences in developmental and temporal trajectories of post‐error slowing.

## STUDY 2

In the second study, we aimed to replicate the Stroop task results and extend the findings to the flanker task. In this task, a target stimulus is flanked by similar stimuli, and cognitive conflict is induced by having the target and flankers pointing to different responses. It could be argued that the flanker task is more a perceptual conflict task than a cognitive conflict task. We were interested in whether the sort of conflict would change the temporal and developmental pattern of post‐error slowing.

### Participants

For the second study, we recruited new participants (55 8‐year‐olds, 39 10‐year‐olds, 29 12‐year‐olds, and 32 young adults). Due to technical problems, we did not obtain Stroop task data from three 8‐year‐olds, one 10‐year‐old, and two adults. After data screening, we applied the following task‐wise exclusion criteria. We excluded the participants who did not commit any error on the critical incongruent trials intended to elicit errors (Stroop task: eleven 8‐year‐olds, eleven 10‐year‐olds, eight 12‐year‐olds, and nine adults; flanker task: eight 8‐year‐olds, ten 10‐year‐olds, eight 12‐year‐olds, and three adults). We also excluded the participants with an accuracy rate under 50% (Stroop task: no one; flanker task: one 8‐year‐old and one adult). This is important because low accuracy rates indicate that they probably did not understand the task, and then they would not be aware of their errors. As error rates influence post‐error slowing (Steinborn et al., 2012), we further excluded the participants with error rates higher than two times the standard deviation (Stroop task: six 8‐year‐olds, and two 10‐year‐olds; flanker task: five 8‐year‐olds, and one 10‐year‐old). This resulted in a more homogenous sample and allowed comparisons across the participants, tasks, and groups. Finally, because we included only correct trials in the analysis of post‐error response times, the participants who committed errors on post‐error trials (i.e., double errors) were also excluded as they did not provide values in those critical cells (Stroop task: three 12‐year‐olds; flanker task: one 10‐year‐old and two 12‐year‐olds). Table 3 presents the demographic characteristics of the final sample.

**TABLE 3:** Study 2: Demographic characteristics of the sample

Age group	Stroop task					Flanker task				
	*M*_Age_	*SD*_Age_	Age range	Female/Male	*n*	*M*_Age_	*SD*_Age_	Age range	Female/Male	*n*
8‐year‐olds	8.1	0.5	7.0–8.9	19/16	35	8.1	0.5	7.0–8.8	17/24	41
10‐year‐olds	10.2	0.3	9.9–10.7	14/11	25	10.2	0.3	9.5–10.9	13/14	27
12‐year‐olds	12.3	0.4	11.7–13.1	11/7	18	12.3	0.5	11.7–13.2	8/11	19
Adults	23	3.6	19.8–37.3	17/4	21	23.1	3.4	19.8–37.3	23/5	28

### Material

The Stroop task was the same as in Study 1. The flanker task was adapted from previous studies (Oeri et al., 2018; Roebers & Kauer, 2009). The stimuli were red fish on blue background facing the left or right side presented on a tablet screen. Example stimuli are presented in Figure 1.

### Procedure

The procedure was the same for children and adults. The participants were tested individually and completed the Stroop task on a tablet and then the flanker task on the same tablet with external response buttons connected to it. The tablets were placed at approximately arm length distance, and the response was given by pressing on the touch screen of the tablet (Stroop task) or by pressing on either the left or right response button (flanker task). The participants were instructed to respond as accurately and fast as possible.

#### Stroop task

Please see Study 1 for a detailed description of this task.

#### Flanker task

The flanker task consisted of two blocks with a fixed block order, which was not repeated. The first block comprised 24 trials, and the second block 120 trials. Seven fish were presented horizontally. Beneath the central fish, at the bottom of the screen, was a shell (cf. Figure 1). In the first block, the participants responded to the side the fish were facing by pressing the left or right response button. For left‐facing fish, the participants had to press the left button with the left hand. For the right‐facing fish, the participants had to press the right button with the right hand. In this purely congruent block, all seven fish were facing the same side. The fish faced 12 times left and 12 times right in random order. The second block was the critical mixed block, in which the central fish (target) sometimes did not face the same side as the other six fish (flankers). These trials were considered incongruent trials. The 24 incongruent trials were determined randomly with replacement and were evenly interspersed among the 96 congruent trials, occurring on every fifth trial. Each trial started with a fixation cross for 250 ms (= response‐stimulus interval) in the middle of the screen, followed by six flankers and the target with a delay of 80 ms. The full array (target and flanker fish) stayed on screen until response. To ensure that the task was clear to the participants, the examiner showed two congruent example trials before the congruent practice block. The congruent and mixed practice blocks consisted of four practice trials. In the case of more than two errors, the examiner explained the task again, and another four practice trials were presented. After successful practice, the respective block started. The mixed block was preceded by four congruent warm‐up trials not included in the analysis.

### Results

#### Stroop task

**FIGURE 3:**
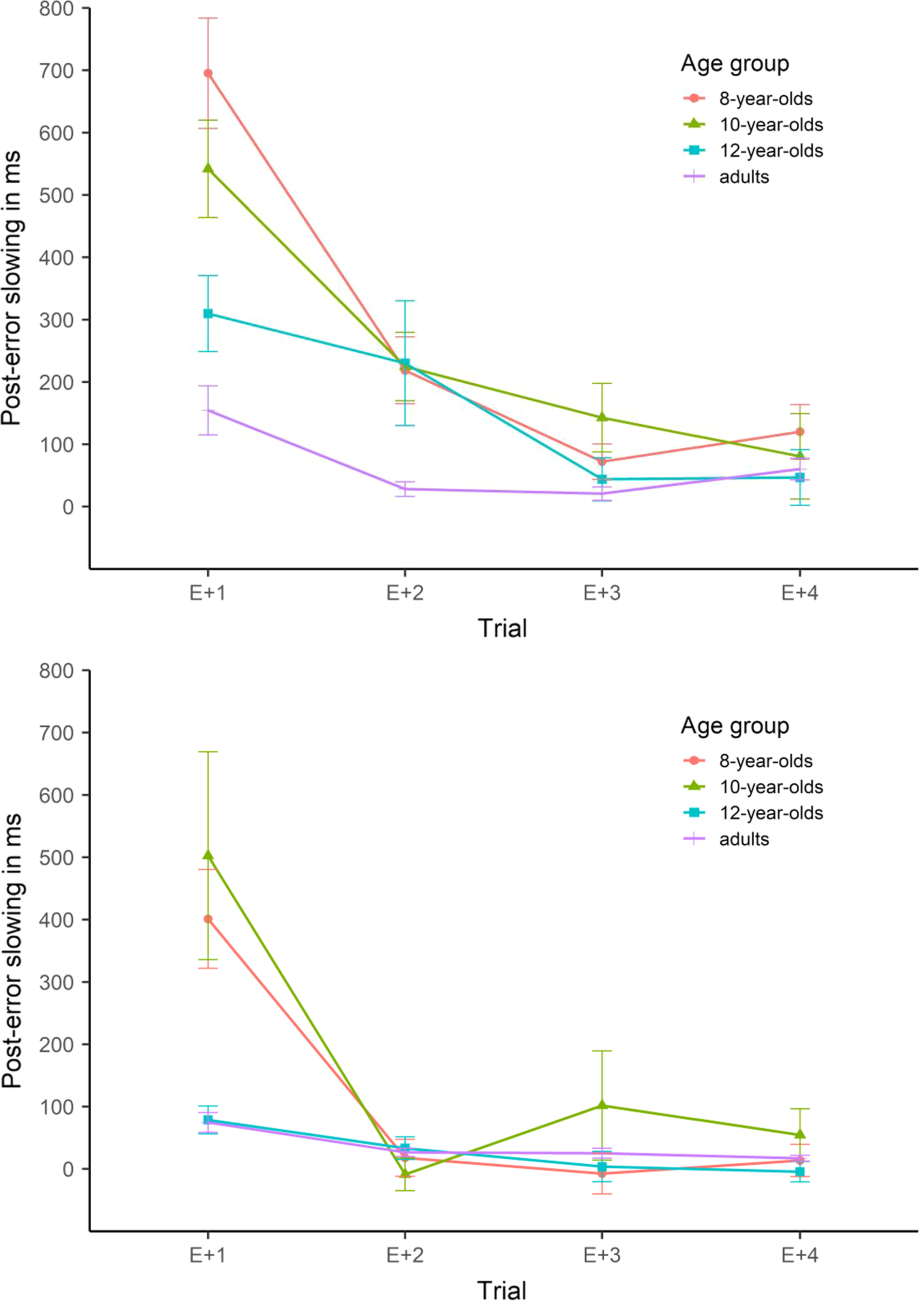
Study 2: Trajectories of post‐error slowing in the Stroop task (upper panel) and flanker task (lower panel). *Note*. Error bars show standard errors

The average number of errors was 3.5 (*SE* = 0.3) for 8‐year‐olds, 2.8 (*SE* = 0.3) for 10‐year‐olds, 2.4 (*SE* = 0.4) for 12‐year‐olds, and 2.2 (*SE* = 0.3) for adults. Table 4 shows response times on correct and incorrect incongruent trials (T + 0) and the subsequent trials (T + 1, T + 2, T + 3, and T + 4). Figure 3 depicts the trajectories of post‐error slowing. The 4 × 4 ANOVA on post‐error slowing revealed a main effect of age group, *F*(3, 95) = 7.05, *p* < .001, $\eta_{p}^{2}$ = .18, and a main effect of trial, *F*(2.38, 225.87) = 36.54, *p* < .001, $\eta_{p}^{2}$ = .28. In line with Study 1, the interaction was also significant, *F*(7.13, 225.87) = 4.24, *p* < .001, $\eta_{p}^{2}$ = .12. To resolve the interaction, we compared the age groups on each trial. Separate one‐way ANOVAs revealed a significant main effect of age group on the first post‐error trial, *F*(3, 95) = 9.56, *p* < .001, $\eta_{p}^{2}$ = .23, but not on the second, third and fourth trials, *F*(3, 95) = 2.40, *p* = .073, $\eta_{p}^{2}$ = .07, *F*(3, 95) = 1.93, *p* = .129, $\eta_{p}^{2}$ = .06, and *F*(3, 95) = 0.45, *p* = .717, $\eta_{p}^{2}$ = .01, respectively. Comparing the age groups on T + 1 revealed an age‐related decrease in post‐error slowing (8 = 10, 8 > 12, 8 > adults; 10 = 12, 10 > adults; 12 = adults).

**TABLE 4:** Study 2: Response times in ms on incongruent conflict trials (T + 0) and subsequent correct congruent trials (T + 1, T + 2, T + 3, and T + 4) as a function of age group and trial type (correctness on T + 0)

Age group	Trial type	T + 0		T + 1		T + 2		T + 3		T + 4	
		*M*	*SE*	*M*	*SE*	*M*	*SE*	*M*	*SE*	*M*	*SE*
*Stroop task*											
8‐year‐olds	Correct	1587	53	1126	52	965	29	968	28	971	34
	Error	1156	63	1821	95	1184	62	1040	42	1091	65
	Difference	−431	**	695	**	219	**	72	*	120	*
10‐year‐olds	Correct	1376	48	952	37	883	34	872	28	852	31
	Error	1152	76	1494	88	1107	60	1015	63	933	74
	Difference	−224	**	542	**	224	**	143	*	81	
12‐year‐olds	Correct	1071	32	722	27	702	19	692	16	686	21
	Error	851	60	1032	56	933	103	736	34	733	38
	Difference	−220	**	310	**	231	*	44		47	
Adults	Correct	807	20	586	10	584	11	566	9	552	7
	Error	645	37	741	45	612	17	587	13	612	22
	Difference	−162	**	155	**	28	*	21	*	60	*
*Flanker task*											
8‐year‐olds	Correct	877	32	779	28	666	20	734	24	656	20
	Error	592	58	1180	88	684	31	726	31	670	26
	Difference	−285	**	401	**	18		−8		14	
10‐year‐olds	Correct	795	44	691	35	616	30	616	27	579	22
	Error	544	94	1193	170	607	32	718	98	634	46
	Difference	−251	**	502	*	−9		102		55	
12‐year‐olds	Correct	570	9	489	13	454	10	473	11	445	13
	Error	346	13	568	24	487	19	477	25	440	16
	Difference	−224	**	79	*	33	*	4		−5	
Adults	Correct	468	5	361	6	346	6	355	6	339	7
	Error	321	6	436	19	372	9	381	9	356	8
	Difference	−147	**	75	**	26	**	26	*	17	**

Note

Significant differences (error – correct) are marked with **p < .001, *p < .05.

#### Flanker task

The average number of errors was 7.8 (*SE* = 0.6) for 8‐year‐olds, 7.4 (*SE* = 0.9) for 10‐year‐olds, 4.2 (*SE* = 0.6) for 12‐year‐olds, and 5.2 (*SE* = 0.7) for adults. Table 4 shows response times on correct and incorrect incongruent trials (T + 0) and the subsequent trials (T + 1, T + 2, T + 3, and T + 4). Figure 3 depicts the trajectories of post‐error slowing. The 4x4 ANOVA on post‐error slowing revealed a marginally significant main effect of age group, *F*(3, 111) = 2.56, *p* = .058, $\eta_{p}^{2}$ = .06. The main effect of trial and the interaction, however, were highly significant, *F*(1.62, 179.62) = 21.44, *p* < .001, $\eta_{p}^{2}$ = .16, and *F*(4.85, 179.62) = 4.46, *p* < .001, $\eta_{p}^{2}$ = .11, respectively. To resolve the interaction, we compared the age groups on each trial. Separate one‐way ANOVAs revealed a significant main effect of age group on the first post‐error trial, *F*(3, 111) = 4.75, *p* = .004, $\eta_{p}^{2}$ = .11, but not on the following trials, *F*(3, 111) = 0.45, *p* = .715, $\eta_{p}^{2}$ = .01, *F*(3, 111) = 1.05, *p* = .372, $\eta_{p}^{2}$ = .03, and *F*(3, 111) = 0.69, *p* = .560, $\eta_{p}^{2}$ = .02, respectively. Comparing the age groups on T + 1 revealed an age‐related decrease in post‐error slowing (8 = 10, 8 = 12, 8 = adults; 10 > 12, 10 > adults; 12 = adults).

### Discussion

In line with Study 1, reliable post‐error slowing emerged in both tasks. The extent and time course of post‐error slowing were comparable across tasks. As hypothesized, post‐error slowing decreased with age. As in Study 1, age‐related differences were most pronounced on the first post‐error trial, with a relatively continuous decrease from 8 years to young adulthood in the Stroop task. In contrast, the flanker task yielded a discontinuous pattern of age‐related differences of slowing on the first post‐error trial. The two younger age groups (8‐ and 10‐year‐olds) showed stronger slowing than the two older age groups (12‐year‐olds and adults). On subsequent trials, post‐error slowing decreased, and so did the age effect in both tasks. This is in line with our hypotheses and the results of Study 1.

## GENERAL DISCUSSION

This study investigated the temporal and developmental trajectories of post‐error slowing across three prominent cognitive conflict tasks. All tasks produced robust post‐error slowing in all age groups. In line with previous research, we found a general decrease in post‐error slowing across trials (Compton et al., 2017; Dubravac et al., 2020; Jentzsch & Dudschig, 2009; Smulders et al., 2016). Slowing on the first post‐error trial consistently decreased from childhood to adulthood (Brewer & Smith, 1989; Carrasco et al., 2013; Carrasco et al., 2013; Fairweather, 1978; Gupta et al., 2009; Schachar et al., 2004; Smulders et al., 2016). Importantly, this age effect diminished on subsequent trials indicating age‐related changes in the temporal trajectory. The younger age groups changed response speed more dramatically, as evidenced by larger slowing on the first post‐error trial with a steeper decrease in slowing on the second trial. This pattern reflects more fine‐tuned adjustments and improving cognitive control from childhood to adulthood (Brewer & Smith, 1989; Diamond, 2013; Luna et al., 2010).

As the Stroop, Simon and flanker tasks differ in the specific kind of conflict, different conflict resolution mechanisms are involved (Egner, 2007). Thus, it was important to subtract conflict related slowing in order to obtain a pure measure of post‐error slowing allowing comparisons between tasks. Compared to the Simon and flanker tasks, post‐error slowing was slightly stronger in the Stroop task. At the same time, error rates were lowest in the Stroop task. As post‐error slowing is found to be strongest with low error rates, the differences in error rates between tasks could explain differences in the extent of post‐error slowing (Notebaert et al., 2009; Steinborn et al., 2012). Apart from this difference, the temporal and developmental trajectories were similar across tasks. In all tasks, post‐error slowing decreased across trials and the age effect was strongest on the first post‐error trial. Considering the different presentation media (tablet/laptop) and response modalities (touchscreen/keyboard/response buttons), the pattern is strikingly consistent across tasks. Together, our results highlight the generality of post‐error slowing when accounting for different error rates, cognitive conflicts, and cognitive control demands (Damaso et al., 2020; Forster & Cho, 2014; Regev & Meiran, 2014).

According to the orienting account of post‐error slowing (Notebaert et al., 2009), infrequent events trigger an orienting response whereby attention is directed towards the infrequent event (i.e., error), resulting in performance slowing on the subsequent trial (i.e., post‐error slowing). Thus, children's stronger slowing on the first post‐error trial likely reflects a stronger orienting response in children as they have more difficulties inhibiting the automatic attention orientation towards the error. This is in accordance with theories of developing inhibitory control (Davidson et al., 2006; Jonkman, 2006; Luna & Sweeney, 2004; Velanova et al., 2008; Williams et al., 1999). In terms of cognitive control, the decreasing variability in response times across post‐error trials suggests more fine‐tuned cognitive control adjustments with age (Botvinick et al., 2001; Brewer & Smith, 1989). This is in accordance with theories of developing abilities to coordinate different cognitive control strategies (Chevalier et al., 2013, 2015). Together, our results suggest that the short‐lived orienting response immediately after the error is more pronounced in children than in adults, while longer lasting and more sophisticated cognitive control adjustments take over on subsequent trials (Botvinick et al., 2001; Notebaert et al., 2009; Wessel, 2018). It is an avenue for future research to pinpoint the developmental trajectories of the different processes underlying post‐error slowing.

In conclusion, this study presents evidence for qualitative developmental changes in post‐error slowing across the Stroop, Simon, and flanker tasks. On the first post‐error trial, slowing decreased with age. This age effect diminished on the following trials suggesting exaggerated behavioural adjustments in children. With age, behavioural adjustments after errors become more fine‐tuned, characterizing qualitative aspects of cognitive control development from 8 years to adulthood. Thus, the fine‐tuning of performance monitoring and cognitive control may underlie age‐related performance improvements in executive function tasks and can be seen as a driving force for developmental progression during elementary school years. The diminishing age effect across trials and the sensitivity to error rates highlight the importance of methodological considerations when investigating post‐error slowing from a developmental perspective.

## CONFLICT OF INTEREST

All authors declare no conflict of interest.

## AUTHOR CONTRIBUTION

**Mirela Dubravac:** Conceptualization (equal); Data curation (equal); Formal analysis (equal); Investigation (equal); Methodology (equal); Project administration (equal); Software (equal); Supervision (equal); Visualization (equal); Writing – original draft (equal); Writing – review & editing (equal). **Claudia M. Roebers:** Conceptualization (equal); Funding acquisition (equal); Methodology (equal); Project administration (equal); Resources (equal); Supervision (equal); Writing – review & editing (equal). **Beat Meier:** Conceptualization (equal); Funding acquisition (equal); Methodology (equal); Project administration (equal); Resources (equal); Supervision (equal); Writing – review & editing (equal).

## Data Availability

The data that support the findings of this study are openly available in Mendeley Data (doi: 10.17632/vd3xhgmfyp.1).

## References

[bjdp12403-bib-0001] Ambrosi, S. , Lemaire, P. , & Blaye, A. (2016). Do young children modulate their cognitive control? Experimental Psychology, 63(2), 117–126. 10.1027/1618-3169/a000320 27221602

[bjdp12403-bib-0002] Anderson, P. (2002). Assessment and development of executive function (EF) during childhood. Child Neuropsychology, 8(2), 71–82. 10.1076/chin.8.2.71.8724 12638061

[bjdp12403-bib-0003] Archibald, S. J. , & Kerns, K. A. (1999). Identification and description of new tests of executive functioning in children. Child Neuropsychology, 5(2), 115–129. 10.1076/chin.5.2.115.3167

[bjdp12403-bib-0004] Botvinick, M. M. , Braver, T. S. , Barch, D. M. , Carter, C. S. , & Cohen, J. D. (2001). Conflict monitoring and cognitive control. Psychological Review, 108(3), 624–652. 10.1037/0033-295X.108.3.624 11488380

[bjdp12403-bib-0005] Brewer, N. , & Smith, G. A. (1989). Developmental changes in processing speed: Influence of speed‐accuracy regulation. Journal of Experimental Psychology: General, 118(3), 298–310. 10.1037/0096-3445.118.3.298

[bjdp12403-bib-0006] Buzzell, G. A. , Beatty, P. J. , Paquette, N. A. , Roberts, D. M. , & McDonald, C. G. (2017). Error‐induced blindness: Error detection leads to impaired sensory processing and lower accuracy at short response–stimulus intervals. Journal of Neuroscience, 37(11), 2895–2903. 10.1523/JNEUROSCI.1202-16.2017 28193697 PMC6596733

[bjdp12403-bib-0007] Carrasco, M. , Harbin, S. M. , Nienhuis, J. K. , Fitzgerald, K. D. , Gehring, W. J. , & Hanna, G. L. (2013). Increased error‐related brain activity in youth with obsessive‐compulsive disorder and unaffected siblings. Depression and Anxiety, 30(1), 39–46. 10.1002/da.22035 23225541

[bjdp12403-bib-0008] Carrasco, M. , Hong, C. , Nienhuis, J. K. , Harbin, S. M. , Fitzgerald, K. D. , Gehring, W. J. , & Hanna, G. L. (2013). Increased error‐related brain activity in youth with obsessive‐compulsive disorder and other anxiety disorders. Neuroscience Letters, 541, 214–218. 10.1016/j.neulet.2013.02.017 23454285 PMC3636987

[bjdp12403-bib-0009] Chevalier, N. (2015). The development of executive function: Toward more optimal coordination of control with age. Child Development Perspectives, 9(4), 239–244. 10.1111/cdep.12138

[bjdp12403-bib-0010] Chevalier, N. , & Blaye, A. (2016). Metacognitive monitoring of executive control engagement during childhood. Child Development, 87(4), 1264–1276. 10.1111/cdev.12537 27084764

[bjdp12403-bib-0011] Chevalier, N. , Huber, K. L. , Wiebe, S. A. , & Espy, K. A. (2013). Qualitative change in executive control during childhood and adulthood. Cognition, 128(1), 1–12. 10.1016/j.cognition.2013.02.012 23562979 PMC4049232

[bjdp12403-bib-0012] Chevalier, N. , Martis, S. B. , Curran, T. , & Munakata, Y. (2015). Metacognitive processes in executive control development: The case of reactive and proactive control. Journal of Cognitive Neuroscience, 27(6), 1125–1136. 10.1162/jocn_a_00782 25603026 PMC4510990

[bjdp12403-bib-0013] Compton, R. J. , Heaton, E. , & Ozer, E. (2017). Intertrial interval duration affects error monitoring. Psychophysiology, 54(8), 1151–1162. 10.1111/psyp.12877 28423188

[bjdp12403-bib-0014] Czernochowski, D. (2014). Conflict monitoring across the life span. Journal of Psychophysiology, 28(3), 124–135. 10.1027/0269-8803/a000120

[bjdp12403-bib-0015] Damaso, K. , Williams, P. , & Heathcote, A. (2020). Evidence for different types of errors being associated with different types of post‐error changes. Psychonomic Bulletin & Review, 27(3), 435–440. 10.3758/s13423-019-01675-w 31907850

[bjdp12403-bib-0016] Danielmeier, C. , & Ullsperger, M. (2011). Post‐error adjustments. Frontiers in Psychology, 2, 1–10. 10.3389/fpsyg.2011.00233 21954390 PMC3173829

[bjdp12403-bib-0017] Davidson, M. C. , Amso, D. , Anderson, L. C. , & Diamond, A. (2006). Development of cognitive control and executive functions from 4 to 13 years: Evidence from manipulations of memory, inhibition, and task switching. Neuropsychologia, 44(11), 2037–2078. 10.1016/j.neuropsychologia.2006.02.006 16580701 PMC1513793

[bjdp12403-bib-0018] Diamond, A. (2013). Executive functions. Annual Review of Psychology, 64(1), 135–168. 10.1146/annurev-psych-113011-143750 PMC4084861 23020641

[bjdp12403-bib-0019] Dubravac, M. , Roebers, C. M. , & Meier, B. (2020). Different temporal dynamics after conflicts and errors in children and adults. PLoS One, 15(8), e0238221. 10.1371/journal.pone.0238221 32866181 PMC7458282

[bjdp12403-bib-0020] Dudschig, C. , & Jentzsch, I. (2009). Speeding before and slowing after errors: Is it all just strategy? Brain Research, 1296, 56–62. 10.1016/j.brainres.2009.08.009 19679114

[bjdp12403-bib-0021] Dutilh, G. , Vandekerckhove, J. , Forstmann, B. U. , Keuleers, E. , Brysbaert, M. , & Wagenmakers, E. J. (2012). Testing theories of post‐error slowing. Attention, Perception, and Psychophysics, 74(2), 454–465. 10.3758/s13414-011-0243-2 PMC3283767 22105857

[bjdp12403-bib-0022] Egner, T. (2007). Congruency sequence effects and cognitive control. Cognitive, Affective, & Behavioral Neuroscience, 7(4), 380–390. 10.3758/CABN.7.4.380 18189011

[bjdp12403-bib-0023] Fairweather, H. (1978). Choice reaction times in children: Error and post‐error responses, and the repetition effect. Journal of Experimental Child Psychology, 26(3), 407–418. 10.1016/0022-0965(78)90121-2

[bjdp12403-bib-0024] Forster, S. E. , & Cho, R. Y. (2014). Context specificity of post‐error and post‐conflict cognitive control adjustments. PLoS One, 9(3), e90281. 10.1371/journal.pone.0090281 24603900 PMC3946012

[bjdp12403-bib-0025] Gratton, G. , Coles, M. G. H. , & Donchin, E. (1992). Optimizing the use of information: Strategic control of activation of responses. Journal of Experimental Psychology: General, 121(4), 480–506. 10.1037/0096-3445.121.4.480 1431740

[bjdp12403-bib-0026] Grundy, J. G. , & Keyvani Chahi, A. (2017). Post‐conflict slowing effects in monolingual and bilingual children. Developmental Science, 20(1), e12488. 10.1111/desc.12488 PMC5199612 27748005

[bjdp12403-bib-0027] Gupta, R. , Kar, B. R. , & Srinivasan, N. (2009). Development of task switching and post‐error‐slowing in children. Behavioral and Brain Functions, 5(1), 38. 10.1186/1744-9081-5-38 19754947 PMC2751760

[bjdp12403-bib-0028] Hämmerer, D. , Müller, V. , & Li, S. C. (2014). Performance monitoring across the lifespan: Still maturing post‐conflict regulation in children and declining task‐set monitoring in older adults. Neuroscience and Biobehavioral Reviews, 46(P1), 105–123. 10.1016/j.neubiorev.2014.06.008 24971826

[bjdp12403-bib-0029] Jentzsch, I. , & Dudschig, C. (2009). Why do we slow down after an error? Mechanisms underlying the effects of posterror slowing. Quarterly Journal of Experimental Psychology, 62(2), 209–218. 10.1080/17470210802240655 18720281

[bjdp12403-bib-0030] Jones, L. B. , Rothbart, M. K. , & Posner, M. I. (2003). Development of executive attention in preschool children. Developmental Science, 6(5), 498–504. 10.1111/1467-7687.00307

[bjdp12403-bib-0031] Jonkman, L. M. (2006). The development of preparation, conflict monitoring and inhibition from early childhood to young adulthood; a Go/Nogo ERP study. Brain Research, 1097(1), 181–193. 10.1016/j.brainres.2006.04.064 16729977

[bjdp12403-bib-0032] King, J. A. , Korb, F. M. , von Cramon, D. Y. , & Ullsperger, M. (2010). Post‐error behavioral adjustments are facilitated by activation and suppression of task‐relevant and task‐irrelevant information processing. Journal of Neuroscience, 30(38), 12759–12769. 10.1523/JNEUROSCI.3274-10.2010 20861380 PMC6633589

[bjdp12403-bib-0033] Laming, D. (1979). Choice reaction performance following an error. Acta Psychologica, 43(3), 199–224. 10.1016/0001-6918(79)90026-X 495175

[bjdp12403-bib-0034] Luna, B. , Padmanabhan, A. , & O’Hearn, K. (2010). What has fMRI told us about the development of cognitive control through adolescence? Brain and Cognition, 72(1), 101–113. 10.1016/j.bandc.2009.08.005 19765880 PMC2815087

[bjdp12403-bib-0035] Luna, B. , & Sweeney, J. A. (2004). The emergence of collaborative brain function: fMRI studies of the development of response inhibition. Annals of the New York Academy of Sciences, 1021(1), 296–309. 10.1196/annals.1308.035 15251900

[bjdp12403-bib-0036] Marco‐Pallarés, J. , Camara, E. , Münte, T. F. , & Rodríguez‐Fornells, A. (2008). Neural mechanisms underlying adaptive actions after slips. Journal of Cognitive Neuroscience, 20(9), 1595–1610. 10.1162/jocn.2008.20117 18345985

[bjdp12403-bib-0037] McDermott, J. M. , Pérez‐Edgar, K. , & Fox, N. A. (2007). Variations of the flanker paradigm: Assessing selective attention in young children. Behavior Research Methods, 39(1), 62–70. 10.3758/BF03192844 17552472

[bjdp12403-bib-0038] Meier, B. , Woodward, T. S. , Rey‐Mermet, A. , & Graf, P. (2009). The bivalency effect in task switching: General and enduring. Canadian Journal of Experimental Psychology, 63(3), 201–210. 10.1037/a0014311 19739903

[bjdp12403-bib-0039] Munakata, Y. , Snyder, H. R. , & Chatham, C. H. (2012). Developing cognitive control: Three key transitions. Current Directions in Psychological Science, 21(2), 71–77. 10.1177/0963721412436807 22711982 PMC3375849

[bjdp12403-bib-0040] Notebaert, W. , Houtman, F. , Opstal, F. V. , Gevers, W. , Fias, W. , & Verguts, T. (2009). Post‐error slowing: An orienting account. Cognition, 111(2), 275–279. 10.1016/j.cognition.2009.02.002 19285310

[bjdp12403-bib-0058] Oeri, N. , Voelke, A. E. , & Roebers, C. M. (2018). Inhibition and behavioral self‐regulation: An inextricably linked couple in preschool years. Cognitive Development, 47, 1–7. 10.1016/j.cogdev.2018.01.004

[bjdp12403-bib-0041] Regev, S. , & Meiran, N. (2014). Post‐error slowing is influenced by cognitive control demand. Acta Psychologica, 152, 10–18. 10.1016/j.actpsy.2014.07.006 25089881

[bjdp12403-bib-0042] Ridderinkhof, K. R. (2002). Micro‐ and macro‐adjustments of task set: Activation and suppression in conflict tasks. Psychological Research Psychologische Forschung, 66(4), 312–323. 10.1007/s00426-002-0104-7 12466928

[bjdp12403-bib-0043] Ridderinkhof, K. R. , & van der Molen, M. W. (1995). A psychophysiological analysis of developmental differences in the ability to resist interference. Child Development, 66(4), 1040–1056. 10.1111/j.1467-8624.1995.tb00921.x

[bjdp12403-bib-0044] Roebers, C. M. (2017). Executive function and metacognition: Towards a unifying framework of cognitive self‐regulation. Developmental Review, 45, 31–51. 10.1016/j.dr.2017.04.001

[bjdp12403-bib-0057] Roebers, C. M. , & Kauer, M. (2009). Motor and cognitive control in a normative sample of 7‐year‐olds. Developmental Science, 12(1), 175–181. 10.1111/j.1467-7687.2008.00755.x 19120425

[bjdp12403-bib-0045] Schachar, R. J. , Chen, S. , Logan, G. D. , Ornstein, T. J. , Crosbie, J. , Ickowicz, A. , & Pakulak, A. (2004). Evidence for an error monitoring deficit in attention deficit hyperactivity disorder. Journal of Abnormal Child Psychology, 32(3), 285–293. 10.1023/B:JACP.0000026142.11217.f2 15228177

[bjdp12403-bib-0046] Schroder, H. S. , Nickels, S. , Cardenas, E. , Breiger, M. , Perlo, S. , & Pizzagalli, D. A. (2020). Optimizing assessments of post‐error slowing: A neurobehavioral investigation of a flanker task. Psychophysiology, 57(2), 10.1111/psyp.13473 PMC6982543 31536142

[bjdp12403-bib-0047] Smulders, S. F. A. , Soetens, E. , & van der Molen, M. W. (2016). What happens when children encounter an error? Brain and Cognition, 104, 34–47. 10.1016/j.bandc.2016.02.004 26914174

[bjdp12403-bib-0048] Smulders, S. F. A. , Soetens, E. L. L. , & van der Molen, M. W. (2018). How do children deal with conflict? A developmental study of sequential conflict modulation. Frontiers in Psychology, 9(766), 1–21. 10.3389/fpsyg.2018.00766 29875718 PMC5974159

[bjdp12403-bib-0049] Steinborn, M. B. , Flehmig, H. C. , Bratzke, D. , & Schröter, H. (2012). Error reactivity in self‐paced performance: Highly‐accurate individuals exhibit largest post‐error slowing. Quarterly Journal of Experimental Psychology, 65(4), 624–631. 10.1080/17470218.2012.660962 22463389

[bjdp12403-bib-0050] Stürmer, B. , Soetens, E. , Leuthold, H. , Schröter, H. , & Sommer, W. (2002). Control over location‐based response activation in the Simon task: Behavioral and electrophysiological evidence. Journal of Experimental Psychology: Human Perception and Performance, 28(6), 1345–1363. 10.1037/0096-1523.28.6.1345 12542132

[bjdp12403-bib-0051] Thaqi, Q. , & Roebers, C. M. (2020). Developmental progression in children’s and adolescents’ cognitive control. Journal of Clinical and Developmental Psychology, 2(2), 48–66. 10.6092/2612-4033/0110-2423

[bjdp12403-bib-0052] Van der Borght, L. , Braem, S. , Stevens, M. , & Notebaert, W. (2016). Keep calm and be patient: The influence of anxiety and time on post‐error adaptations. Acta Psychologica, 164, 34–38. 10.1016/j.actpsy.2015.12.007 26720098

[bjdp12403-bib-0053] Velanova, K. , Wheeler, M. E. , & Luna, B. (2008). Maturational changes in anterior cingulate and frontoparietal recruitment support the development of error processing and inhibitory control. Cerebral Cortex, 18(11), 2505–2522. 10.1093/cercor/bhn012 18281300 PMC2733315

[bjdp12403-bib-0054] Wessel, J. R. (2018). An adaptive orienting theory of error processing. Psychophysiology, 55(3), e13041. 10.1111/psyp.13041 29226960

[bjdp12403-bib-0055] Williams, B. R. , Ponesse, J. S. , Schachar, R. J. , Logan, G. D. , & Tannock, R. (1999). Development of inhibitory control across the life span. Developmental Psychology, 35(1), 205–213. 10.1037/0012-1649.35.1.205 9923475

[bjdp12403-bib-0056] Woodward, T. S. , Meier, B. , Tipper, C. , & Graf, P. (2003). Bivalency is costly: Bivalent stimuli elicit cautious responding. Experimental Psychology, 50(4), 233–238. 10.1027//1618-3169.50.4.233 14587170

