# Supplementary material for: Age‐related qualitative differences in post‐error cognitive control adjustments
Source: Br J Dev Psychol. 2022 Jan 18;40(2):287–305. doi: 10.1111/bjdp.12403 (PMC9306937; doi:10.1111/bjdp.12403)
Supplement: Supplementary file 1 [file BJDP-40-287-s001.docx]

Supplementary Material

**Age-related qualitative differences in post-error cognitive control adjustments**

# Excluded Participants

Response times on incongruent trials of participants who were excluded because they did not commit any error on incongruent trials.

## Study 1

| Age group | Stroop task | | |  | Simon task | | |
| --- | --- | --- | --- | --- | --- | --- | --- |
|  | *M* | *SE* | n |  | *M* | *SE* | n |
| 8-year-olds | 1366 | 52 | 9 |  | 976 | 48 | 3 |
| 10-year-olds | 1180 | 54 | 7 |  | 778 | 35 | 6 |
| 12-year-olds | 1087 | 21 | 11 |  | 629 | 34 | 6 |
| adults | 852 | 28 | 17 |  | 493 | 15 | 5 |

## Study 2

| Age group | Stroop task | | |  | Flanker task | | |
| --- | --- | --- | --- | --- | --- | --- | --- |
|  | *M* | *SE* | n |  | *M* | *SE* | n |
| 8-year-olds | 1602 | 84 | 11 |  | 1070 | 121 | 8 |
| 10-year-olds | 1290 | 33 | 11 |  | 765 | 62 | 10 |
| 12-year-olds | 1105 | 56 | 8 |  | 580 | 15 | 8 |
| adults | 833 | 23 | 9 |  | 512 | 17 | 3 |

# Congruency effect

The Stroop, Simon, and flanker tasks elicit cognitive conflict on incongruent trials, where irrelevant stimulus dimensions (in the Stroop and Simon tasks) or irrelevant distractors (in the flanker task) trigger a prepotent but incorrect response that has to be inhibited to respond correctly. In contrast, congruent trials involve no conflict as the relevant and irrelevant stimulus dimensions and distractors call for the same response. Incongruent trials are thus expected to increase error rates and response times relative to congruent trials (i.e., congruency effect). We expected substantial congruency effects in all tasks and an age-related decrease in the congruency effect as inhibitory control improves with age (Ridderinkhof & van der Molen, 1995).

To test the expected **congruency effect** of lower accuracy rates and higher response times on incongruent compared to congruent trials in all four age groups, we conducted two separate 2x4 mixed ANOVAs with trial type and age group as factors. To resolve significant interactions, we computed the difference between the congruent and incongruent conditions (i.e., the congruency effect) and compared the age groups. We applied the Bonferroni correction for multiple comparisons.

# Study 1

## Stroop task

Figure 1 depicts the distribution of error rates, and Figure 2 depicts mean response times for congruent and incongruent trials. The ANOVA on **accuracy** revealed a main effect of trial type, *F*(1, 120) = 356.80, *p* < .001, $\eta_{p}^{2}$ = .75, but no main effect of age group, *F*(3, 120) = 1.93, *p* = .128, $\eta_{p}^{2}$ = .05, and no interaction, *F*(3, 120) = 1.00, *p* = .396, $\eta_{p}^{2}$ = .02. The lower accuracy rates on incongruent trials (*M* = 91.6%, *SE* = 0.4%) compared to congruent trials (*M* = 99.6%, *SE* = 0.1%) indicates that the manipulation of inducing errors by incongruent trials was successful. The ANOVA on **response times** revealed a main effect of trial type, *F*(1, 120) = 1017.98, *p* < .001, $\eta_{p}^{2}$ = .89, and a main effect of age group, *F*(3, 120) = 66.86, *p* < .001, $\eta_{p}^{2}$ = .63. The interaction was significant, *F*(3, 120) = 22.33, *p* < .001, $\eta_{p}^{2}$ = .36, indicating a decrease in the congruency effect with age (8 = 10, 8 > 12, 8 > adults, 10 = 12, 10 > adults, 12 > adults).

## Simon task

Figure 3 depicts the distribution of error rates, and Figure 4 depicts mean response times for congruent and incongruent trials. The ANOVA on **accuracy** revealed a main effect of trial type, *F*(1, 147) = 278.39, *p* < .001, $\eta_{p}^{2}$ = .65, but no main effect of age group, *F*(3, 147) = 1.96, *p* = .122, $\eta_{p}^{2}$ = .04, and no interaction, *F*(3, 147) = 0.58, *p* = .630, $\eta_{p}^{2}$ = .01. This is consistent with the results of the Stroop task. The lower accuracy rates on incongruent trials (*M* = 85.1 %, *SE* = 0.8%) compared to congruent trials (*M* = 98.0%, *SE* = 0.2%) indicates that the manipulation of inducing errors by incongruent trials was successful. The ANOVA on **response times** revealed again a main effect of trial type, *F*(1, 147) = 604.96, *p* < .001, $\eta_{p}^{2}$ = .80, and a main effect of age group, *F*(3, 147) = 121.04, *p* < .001, $\eta_{p}^{2}$ = .71. Also consistent with the results of the Stroop task, the interaction was significant, *F*(3, 147) = 17.14, *p* < .001, $\eta_{p}^{2}$ = .26, indicating a decreasing congruency effect with age (8 = 10, 8 > 12, 8 > adults, 10 > 12, 10 > adults, 12 = adults).

## Discussion

In both tasks, incongruent trials elicited a cognitive conflict resulting in a congruency effect of more errors and higher response times on incongruent trials. The interaction between congruency and age group on response times indicated a relatively gradual decrease in the congruency effect across age groups. This is in line with our hypothesis of an age-related decrease in the congruency effect.

# Study 2

## Stroop task

Figure 5 depicts the distribution of error rates, and Figure 6 depicts mean response times for congruent and incongruent trials. The ANOVA on **accuracy** revealed a main effect of trial type, *F*(1, 95) = 239.09, *p* < .001, $\eta_{p}^{2}$ = .72. In contrast to Study 1, the main effect of age group was significant, *F*(3, 95) = 3.65, *p* = .015, $\eta_{p}^{2}$ = .10, as well as the interaction, *F*(3, 95) = 2.87, *p* = .040, $\eta_{p}^{2}$ = .08. Post-hoc tests on the congruency effect, however, yielded no significant differences between age groups. The ANOVA on **response times** revealed again a main effect of trial type, *F*(1, 95) = 785.93, *p* < .001, $\eta_{p}^{2}$ = .89, as well as a main effect of age group, *F*(3, 95) = 55.50, *p* < .001, $\eta_{p}^{2}$ = .64. In line with Study 1, the interaction was significant, *F*(3, 95) = 28.14, *p* < .001, $\eta_{p}^{2}$ = .47, indicating a decrease in the congruency effect with age (8 = 10, 8 > 12, 8> adults, 10 = 12, 10 > adults, 12 > adults).

## Flanker task

Figure 7 depicts the distribution of error rates, and Figure 8 depicts mean response times for congruent and incongruent trials. The ANOVA on **accuracy** revealed a main effect of trial type, *F*(1, 111) = 159.34, *p* < .001, $\eta_{p}^{2}$ = .59, but no main effect of age group, *F*(3, 111) = 1.87, *p* = .139, $\eta_{p}^{2}$ = .05. The interaction was significant, *F*(3, 111) = 12.89, *p* < .001, $\eta_{p}^{2}$ = .26. The lower accuracy rates on incongruent trials (*M* = 86.0 %, *SE* = 0.9%) compared to congruent trials (*M* = 96.8%, *SE* = 0.3%) indicates that the manipulation of inducing errors by incongruent trials was successful. Surprisingly, the congruency effect was largest in adults (8 = 10 = 12 < adults). The accuracy differences between congruent and incongruent trials in percent points were: 7.0 in 8-year-olds, 7.2 in 10-year-olds, 10.7 in 12-year-olds, and 19.9 in adults. Accuracy rates for congruent trials were as follows: 8-year-olds (*M* = 94.9%, *SE* = 0.5%), 10-year-olds (*M* = 95.3%, *SE* = 0.7%), 12-year-olds (*M* = 98.6%, *SE* = 0.5%), and adults (*M* = 99.6%, *SE* = 0.2%). Accuracy rates for incongruent trials were as follows: 8-year-olds (*M* = 87.9%, *SE* = 1.2%), 10-year-olds (*M* = 88.1%, *SE* = 1.8%), 12-year-olds (*M* = 87.9%, *SE* = 1.4%), and adults (*M* = 79.8%, *SE* = 2.4%). The ANOVA on **response times** revealed a main effect of trial type, *F*(1, 111) = 183.21, *p* < .001, $\eta_{p}^{2}$ = .62, age group, *F*(3, 111) = 64.73, *p* < .001, $\eta_{p}^{2}$ = .64, and a significant interaction, *F*(3, 111) = 3.80, *p* = .012, $\eta_{p}^{2}$ = .09. Post-hoc tests, however, yielded no significant differences in the congruency effect between age groups.

## Discussion

In line with Study 1, we found a congruency effect of lower accuracy rates and higher response times on incongruent trials in both tasks. In line with previous research, the congruency effect on response times decreased with age in the Stroop task (Davidson et al., 2006; Erb & Marcovitch, 2018, 2019; Ridderinkhof & van der Molen, 1995). Complementing Study 1, the interaction between congruency and age group was not only significant for response times but also for accuracy rates. While the age effect on congruency was negligible in the Stroop task, an unexpected pattern emerged in the flanker task. Surprisingly, the congruency effect was largest in adults. While accuracy rates on congruent trials slightly increased with age, accuracy rates on incongruent trials decreased dramatically. Adults’ accuracy rates were almost ten percent points lower than children’s accuracy rates. The distribution of error rates suggests that outliers did not drive this effect (cf. Fig. 7). In fact, the error distribution in the flanker task was even more balanced than in the other tasks. Thus, it is safe to conclude that the flanker task was more difficult for adults than for children.

It seems that younger participants were better at focusing on the central fish and ignoring the incongruent flanking fish. This age pattern contrasts with the assumption of better inhibitory control with age (Davidson et al., 2006; Diamond, 2013; Luna & Sweeney, 2004). Speculating that inhibitory control plays a minor role in this task, the transition from analytic to holistic perceptual processing may explain these counterintuitive results. Analytic processing refers to perceiving specific and individual features of a visual array separately. In contrast, holistic processing refers to perceiving the individual features and their spatial relations as an integrated whole. In one study, a perceptual matching task was used to assess the degree to which children and adults perceive faces analytically versus holistically (Joseph et al., 2015). The authors manipulated the similarity of features and spatial relations and assessed participants’ response times when discriminating pairs of faces and houses. They found that younger children (6-8 years) showed analytical processing, older children (9-11 years) showed an intermediate pattern, while adults showed holistic processing (Joseph et al., 2015). In the flanker task, holistic processing of the whole array of fish increases the conflict, while analytical processing allows focusing more on the task-relevant, central fish. Different preferences in processing style may thus lead to differences in performance.

# References

Davidson, M. C., Amso, D., Anderson, L. C., & Diamond, A. (2006). Development of cognitive control and executive functions from 4 to 13 years: Evidence from manipulations of memory, inhibition, and task switching. *Neuropsychologia*, *44*(11), 2037–2078. https://doi.org/10.1016/j.neuropsychologia.2006.02.006

Diamond, A. (2013). Executive functions. *Annual Review of Psychology*, *64*(1), 135–168. https://doi.org/10.1146/annurev-psych-113011-143750

Erb, C. D., & Marcovitch, S. (2018). Deconstructing the Gratton effect: Targeting dissociable trial sequence effects in children, pre-adolescents, and adults. *Cognition*, *179*, 150–162. https://doi.org/10.1016/j.cognition.2018.06.007

Erb, C. D., & Marcovitch, S. (2019). Tracking the within‐trial, cross‐trial, and developmental dynamics of cognitive control: Evidence from the Simon task. *Child Development*, *90*(6), 831–848. https://doi.org/10.1111/cdev.13111

Joseph, J. E., DiBartolo, M. D., & Bhatt, R. S. (2015). Developmental changes in analytic and holistic processes in face perception. *Frontiers in Psychology*, *6*(1165). https://doi.org/10.3389/fpsyg.2015.01165

Luna, B., & Sweeney, J. A. (2004). The emergence of collaborative brain function: fMRI studies of the development of response inhibition. *Annals of the New York Academy of Sciences*, *1021*(1), 296–309. https://doi.org/10.1196/annals.1308.035

Ridderinkhof, K. R., & van der Molen, M. W. (1995). A psychophysiological analysis of developmental differences in the ability to resist interference. *Child Development*, *66*(4), 1040–1056. https://doi.org/10.1111/j.1467-8624.1995.tb00921.x

# Figures

## Figure 1


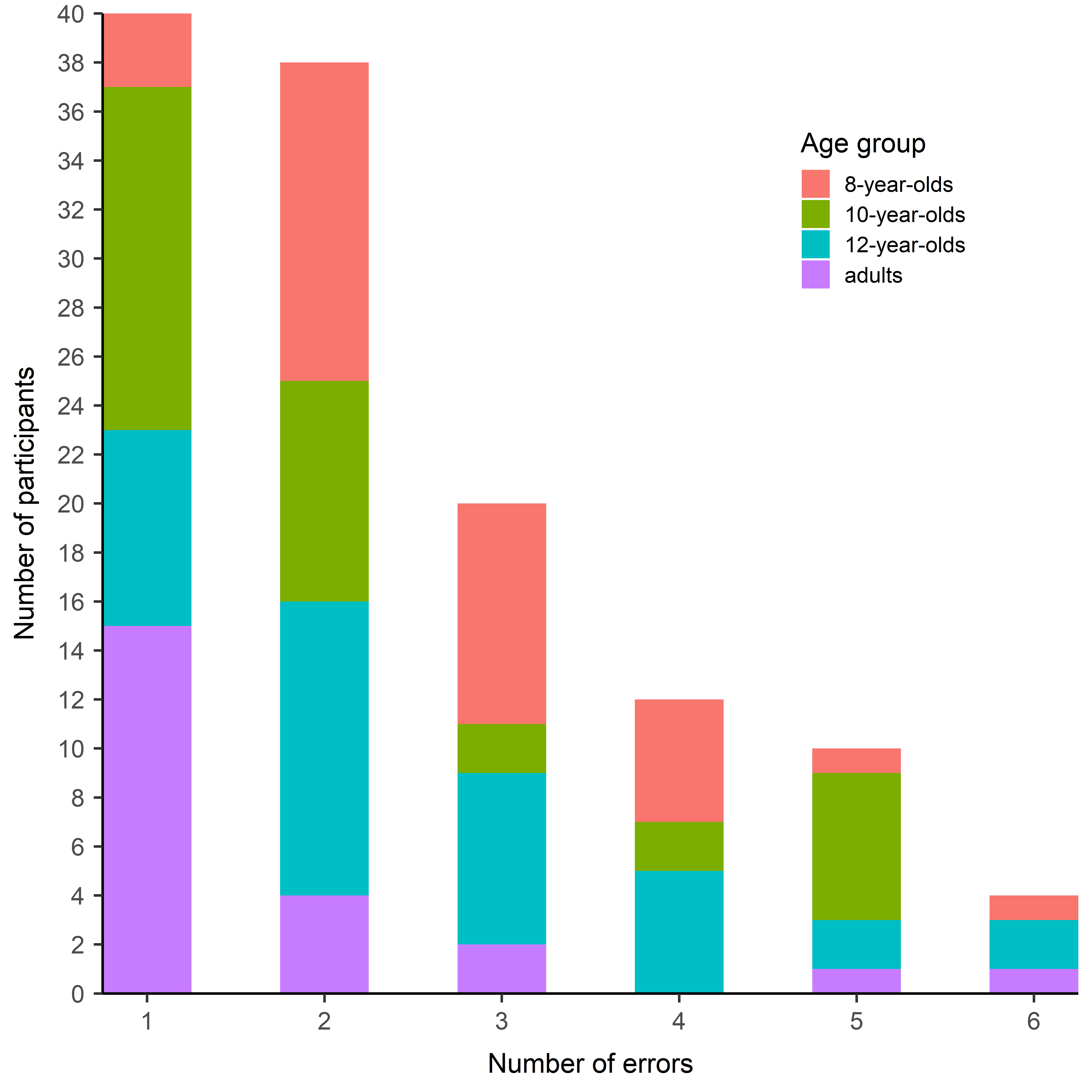
*Study 1: Distribution of error rates in the Stroop task*

## Figure 2


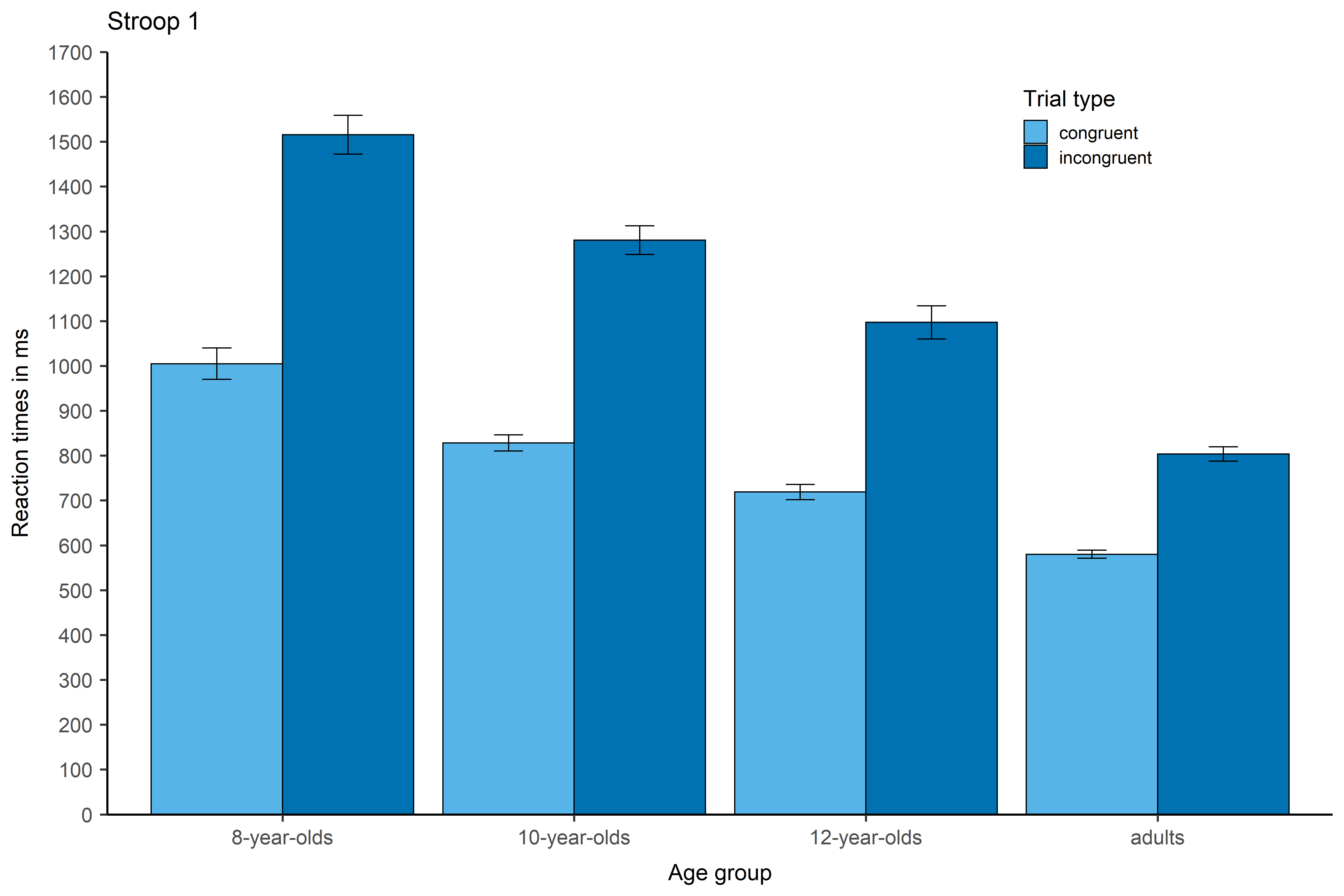
*Study 1: Congruency effect in the Stroop task*

*Note*. Depicted are mean response times on correct congruent and correct incongruent trials per age group. Error bars show standard errors.

## Figure 3


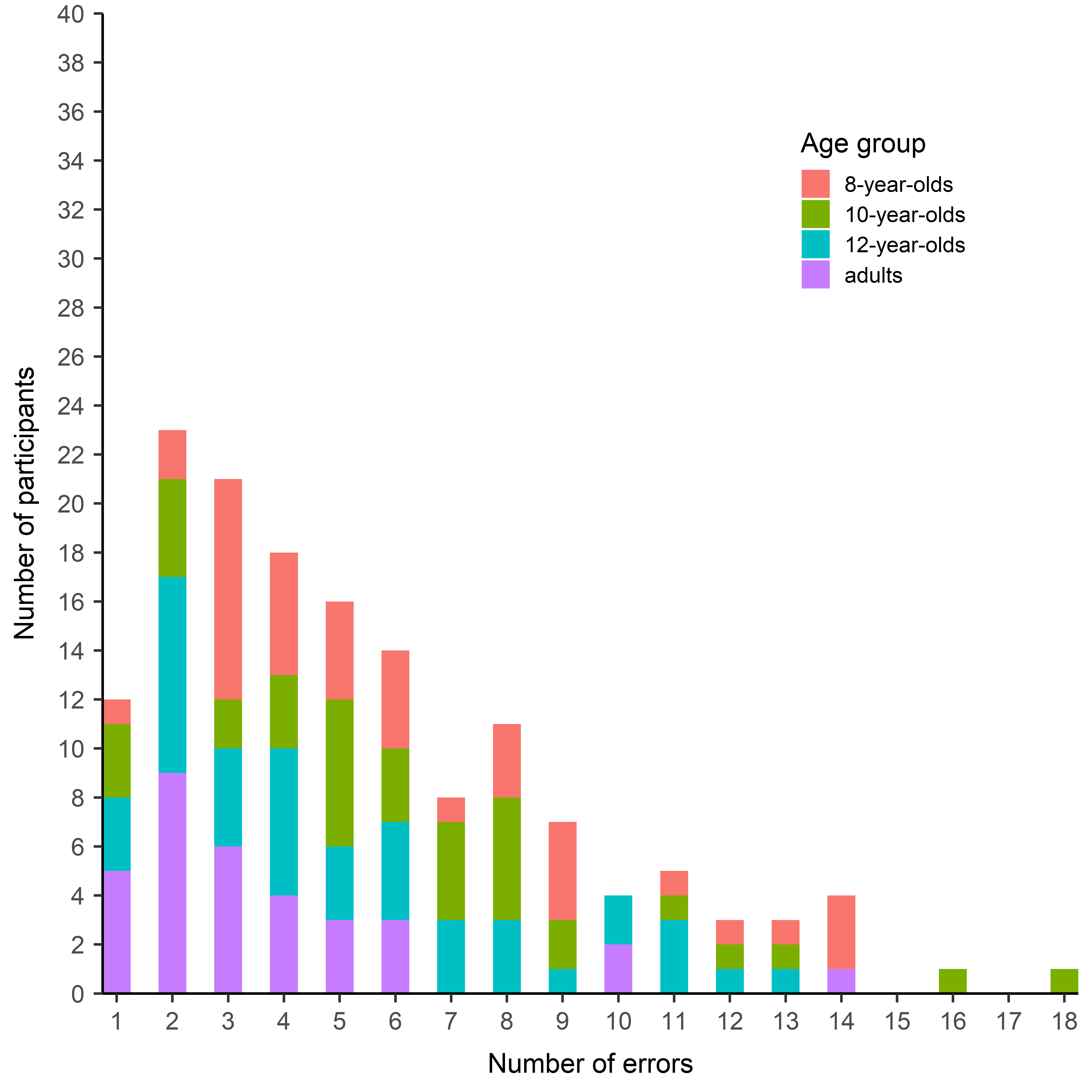
*Study 1: Distribution of error rates in the Simon task*

## Figure 4


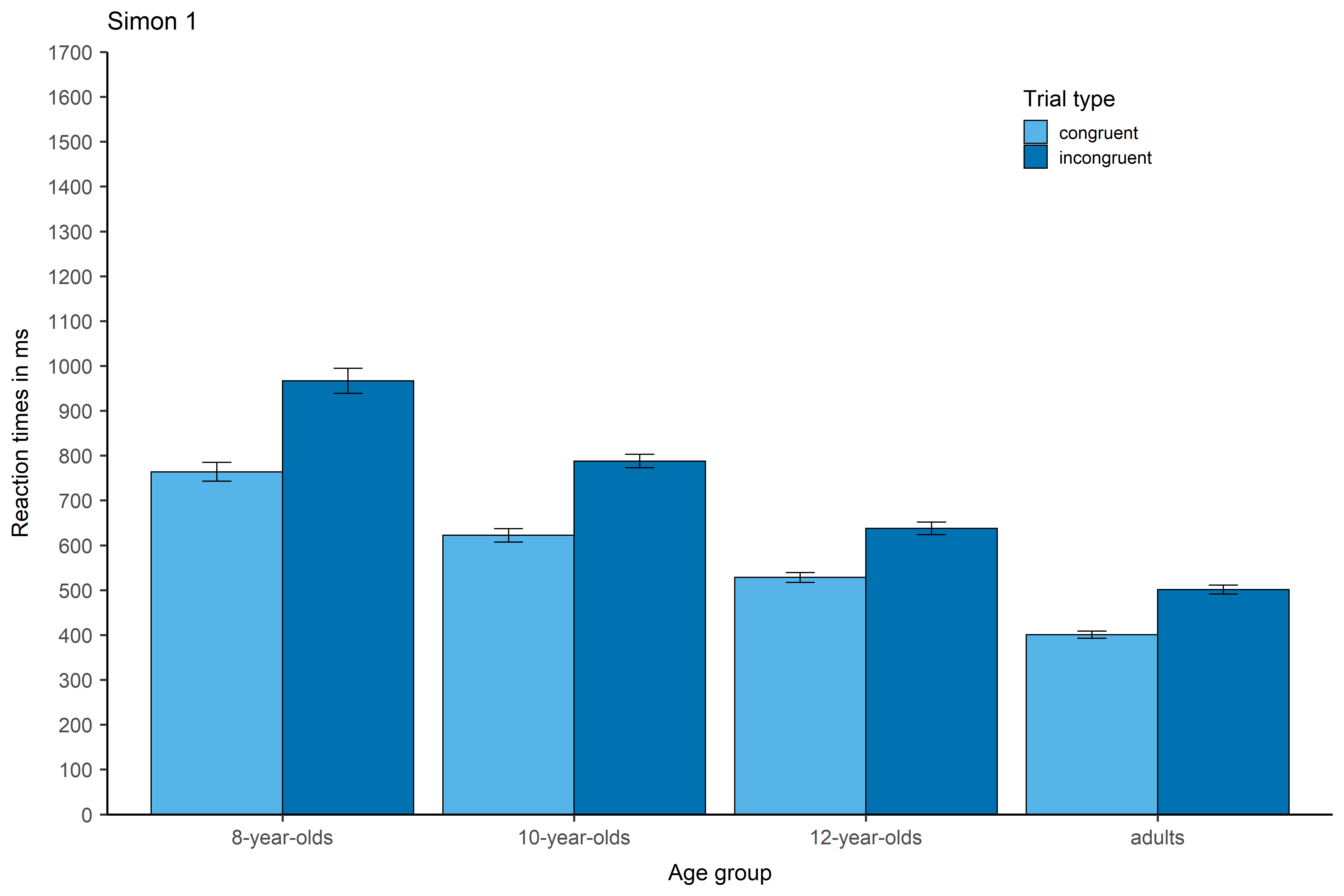
*Study 1: Congruency effect in the Simon task*

*Note*. Depicted are mean response times on correct congruent and correct incongruent trials per age group. Error bars show standard errors.

## Figure 5


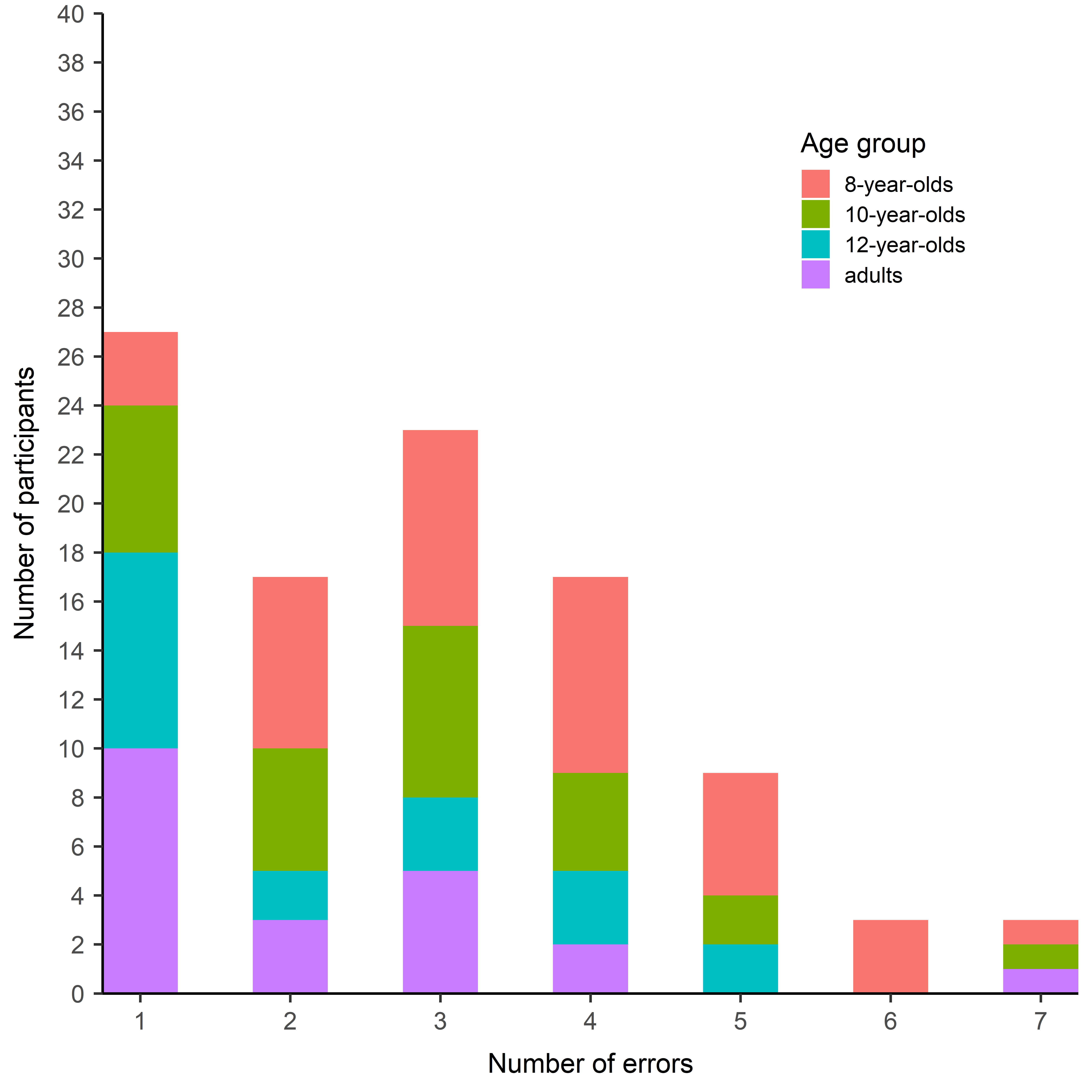
*Study 2: Distribution of error rates in the Stroop task*

## Figure 6


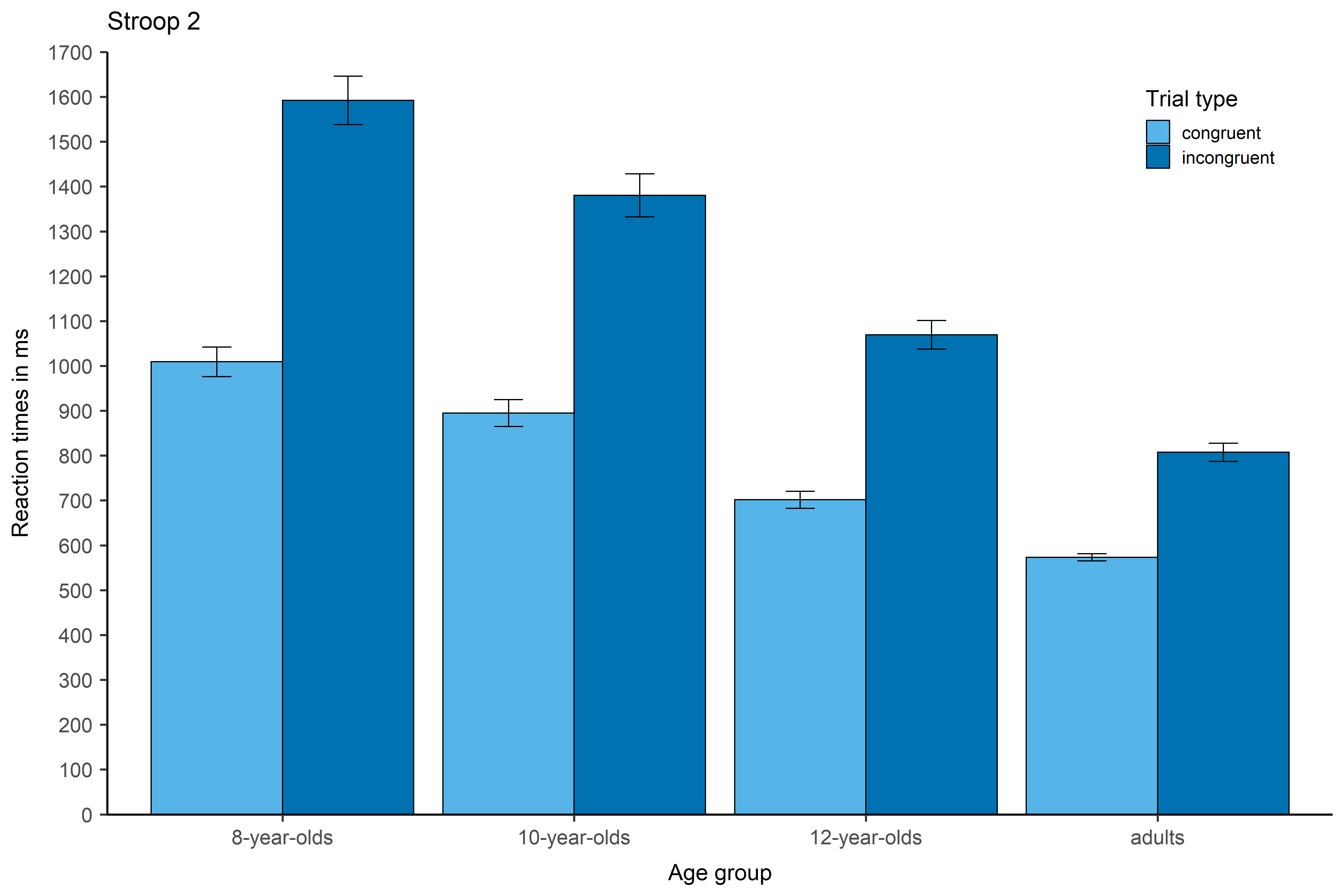
*Study 2: Congruency effect in the Stroop task*

*Note*. Depicted are mean response times on correct congruent and correct incongruent trials per age group. Error bars show standard errors.

## Figure 7


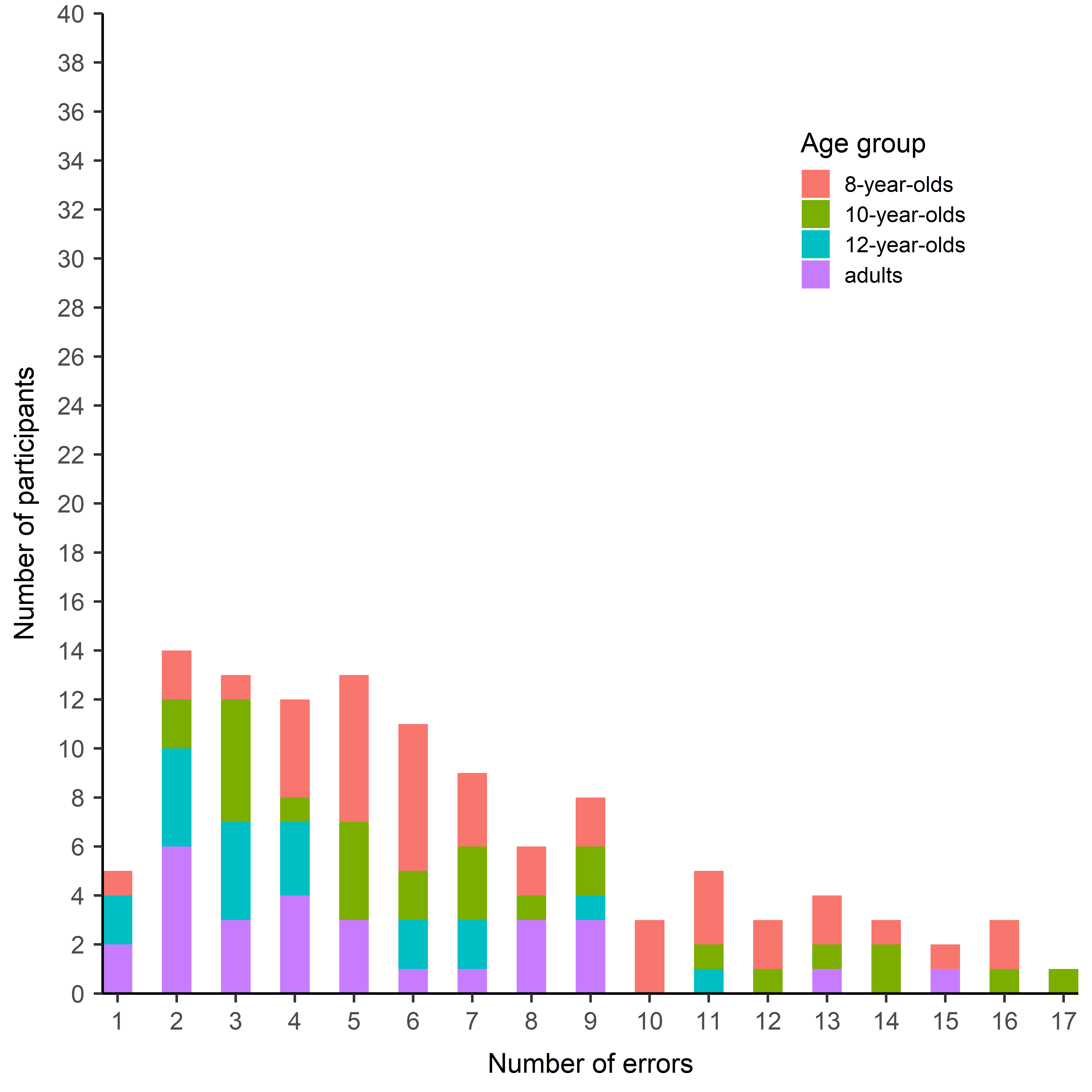
*Study 2: Distribution of error rates in the flanker task*

## Figure 8


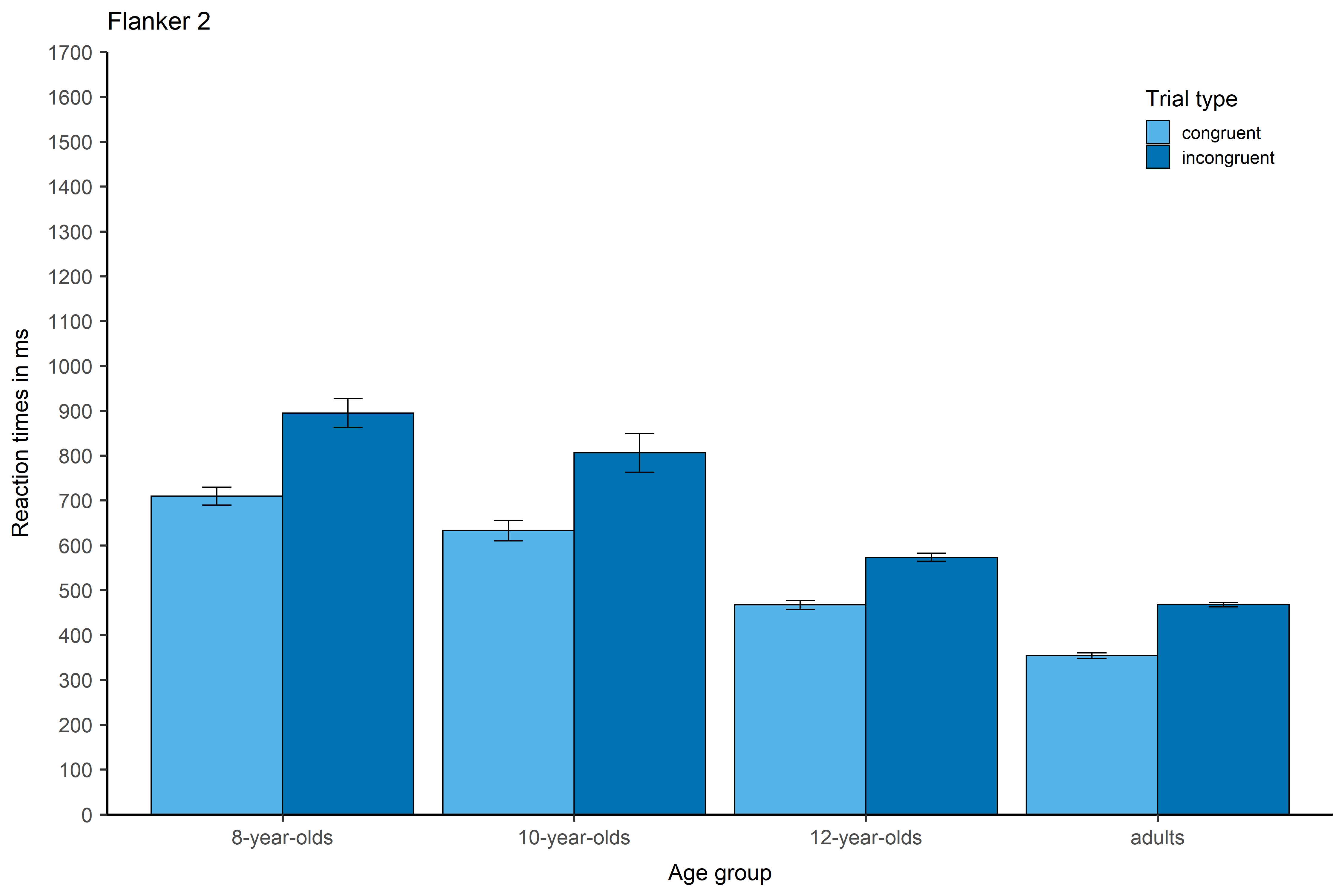
*Study 2: Congruency effect in the flanker task*

*Note*. Depicted are mean response times on correct congruent and correct incongruent trials per age group. Error bars show standard errors.
